# PRISM: Feature-Guided Hierarchical Inpainting for Dual-Band Infrared Defective Pixel Clusters

**DOI:** 10.3390/s26175417

**Published:** 2026-08-27

**Authors:** Xu Zhao, Jinxin Wang, Xiaoli Xi, Fang Li, Yingqiang Xu, Hongyue Hao, Dongwei Jiang, Dongmei Li

**Affiliations:** 1 Optoelectronic System Laboratory, Institute of Semiconductors, Chinese Academy of Sciences, Beijing 100083, China; 2 College of Materials Science and Opto-Electronic Technology, University of Chinese Academy of Sciences, Beijing 100049, China; 3 State Key Laboratory for Superlattices and Microstructures, Institute of Semiconductors, Chinese Academy of Sciences, Beijing 100083, China

**Keywords:** infrared image restoration, exemplar-based inpainting, dual-band imager, defective pixel clusters, type-II superlattice

## Abstract

This paper presents a feature-level defective pixel cluster (DPC) correction method specifically designed for dual-band infrared detectors. Due to inherent manufacturing limitations, antimonide-based type-II superlattice (T2SL) focal plane arrays (FPAs) commonly suffer from DPC issues. Existing DPC correction methods often fail to preserve dual-band imaging characteristics, leading to texture distortion and incomplete structure recovery. Inspired by infrared imaging mechanisms and image inpainting theory, we propose PRISM, a patch-based reconstruction framework that leverages inter-band structure migration with hierarchical feature decomposition, decoupling dual-band images into micro-textures, edge gradients, and spatial relationships. Leveraging this multi-level feature representation, we extract prior information to guide DPC correction. The correction process is formulated as a “structure-to-pixel” optimization problem, where an improved feature-guided patch search strategy effectively combines structure completion with texture reconstruction. Experimental results demonstrate that the proposed method not only recovers image content effectively but also faithfully preserves band-specific imaging characteristics. Furthermore, to facilitate quantitative evaluation, we construct a benchmark dataset containing simulated DPCs with corresponding ground truth. Comparative experiments on both our self-constructed dataset and public multimodal benchmarks confirm that PRISM achieves higher PSNR, SSIM, VIF, and CC metrics than state-of-the-art multimodal inpainting methods.

## 1. Introduction

The fourth-generation dual-band infrared focal plane array (FPA) represents a key technology in multi-spectral detection systems, particularly those based on antimonide type-II superlattice (T2SL) materials [1]. By exploiting the radiance differences between targets and backgrounds across different spectral bands, dual-band detectors have demonstrated significant potential in applications such as person re-identification [2], image fusion [3], and camouflaged object detection [4]. In particular, short-wave/mid-wave infrared (SWIR/MWIR) dual-band T2SL detectors can simultaneously capture reflected solar radiation and thermal self-emission information. This dual-band imaging capability ensures excellent temporal synchronization and strict spatial pixel-wise registration between the two bands [5].

**Figure 1:**
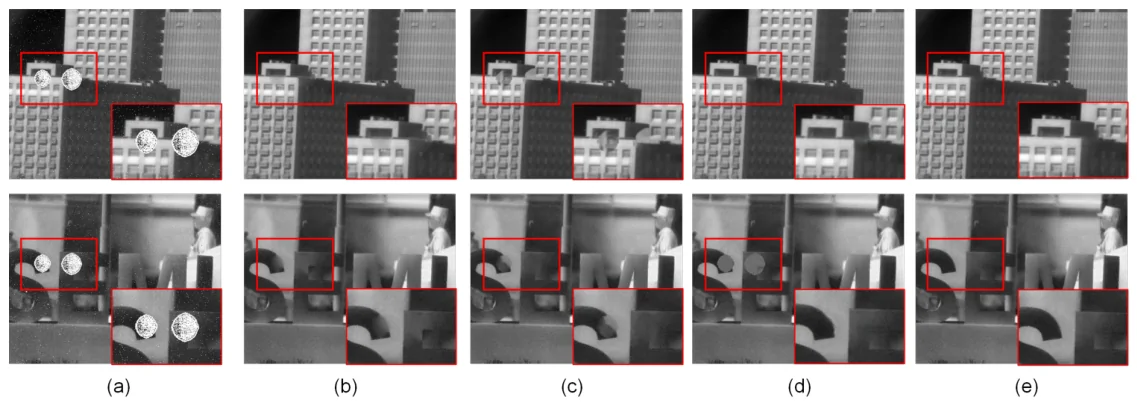
Qualitative comparison on SWIR/MWIR T2SL FPA imagery. (**a**) Raw Images with DPC; (**b**) DIP [18]; (**c**) Huang et al. [17]; (**d**) PEIPNet [31]; (**e**) Proposed (PRISM).

Despite their promising applications, dual-band detectors face significant manufacturing challenges [6]. Compared with mature single-band technologies, dual-band FPAs are highly sensitive to lattice mismatch and stress accumulation. Additionally, defects frequently arise during mesa etching and passivation processes [7,8], leading to high defect densities in epitaxial layers that form large-area defective pixel clusters (DPCs). As illustrated in Figure 1a, these cluster-like defects compromise image integrity and continuity. Such degradation undermines the advantages of dual-band information fusion, necessitating algorithmic DPC correction before practical deployment.

The existing widely used correction methods [9,10,11,12,13,14,15] are primarily designed for isolated point defects and small clusters; they often fail when confronted with large-area DPCs due to the severe insufficiency of valid neighborhood information. More importantly, existing single-band and dual-band correction methods fail to preserve both structural continuity and band-specific radiometric fidelity simultaneously: single-band approaches [16,17] cannot exploit complementary cross-band information, while dual-band methods such as DSL/DIP [18] rely on direct pixel-wise intensity mapping that breaks down when the two bands capture fundamentally different physical signals. This motivates the need for feature-level cross-band transfer rather than pixel-level intensity mapping. To address large missing regions, patch-based image inpainting methods [16,17,19,20,21,22,23,24] have been extensively studied, achieving promising results in reconstructing regular textures and background details. Recently, deep learning approaches have significantly advanced inpainting technology by learning high-level semantic representations from large-scale datasets [25,26,27,28,29,30,31,32,33,34,35]. From early context encoders to modern networks employing context attention mechanisms and Transformer architectures, deep models excel at capturing global consistency and synthesizing complex local textures, effectively reducing the visual saliency of DPCs and generating more natural restoration results. Furthermore, for multi-band detectors, Nguyen et al. [18] explored cross-spectral information fusion, proposing dual-band statistical lookup table (DSL) and dual-band image painting (DIP) methods based on inter-band correlations.

However, the aforementioned methods exhibit evident limitations when addressing specific infrared compensation tasks. As shown in Figure 1b–d, we compare three representative methods—the spectral-correlation-based DIP [18], the patch-based method of Huang et al. [17], and the deep learning-based PEIPNet [31]—on actual restoration results. Since these methods were not specifically designed for SWIR/MWIR dual-band detector compensation tasks, they cannot effectively utilize the physical consistency of infrared cross-bands, resulting in failure to accurately recover missing structures and ultimately suboptimal restoration performance.

To overcome these limitations, we propose PRISM (Patch-based Reconstruction through Inter-band Structure Migration), which is a novel method based on cross-band structure migration for dual-band infrared DPC inpainting. PRISM differs from prior methods in three fundamental aspects. Unlike DIP [18], which assumes direct pixel-wise intensity mapping between bands and therefore fails when SWIR reflection and MWIR thermal emission capture physically different signals, PRISM operates at the feature level—transferring structural and textural cues rather than raw intensities. Unlike the work of Huang et al. [17], which exploits planar regularity within a single band and cannot exploit cross-band complementary information, PRISM leverages the pixel-wise registration property of dual-band detectors to transfer reliable geometric priors from the reference band. Unlike PEIPNet [31] and other deep learning approaches that require large-scale training data and may produce structures inconsistent with target-band radiometric properties, PRISM is training-free and physically interpretable, strictly preserving band-specific imaging characteristics. The core philosophy lies in leveraging the multi-level characteristics of image features together with a coarse-to-fine iterative reconstruction strategy. To achieve precise cross-band information transfer, we design a Cross-band Multi-level Feature Analysis (CMFA) module that decomposes infrared images into independent spatial layout, edge structure, and texture feature components. This decomposition enables the estimation of local inter-band relationships during patch matching. By exploiting the invariance of scene spatial features and the correlation of structural features across different spectra, our method first reconstructs the structural backbone, which then provides core guidance for subsequent DPC correction. Subsequently, we formulate the texture recovery process as a maximum a posteriori (MAP) inference problem. Our method explicitly distinguishes between expansion into known regions and inference within unknown regions, thereby naturally handling the reliability differences among information sources. Finally, by leveraging previously reconstructed spatial and structural information to constrain the patch search process, we further optimize texture reconstruction accuracy.

The objectives of this paper are threefold: (i) to correct large-area DPCs that existing single-band and dual-band methods fail to handle; (ii) to preserve dual-band fidelity, ensuring that band-specific radiometric characteristics are not contaminated by reference-band features; and (iii) to enable quantitative evaluation through a constructed benchmark dataset with ground truth.

The main contributions of this paper are summarized as follows:

- **Cross-band Multi-level Feature Analysis:** We propose a robust decomposition method that decomposes dual-band infrared images into spatial, edge, and texture multi-level feature representations, extracting common features while preserving band-specific differential characteristics.
- **Multi-level Feature-Guided Reconstruction (MFGR):** We design a cascaded architecture integrating structure completion, texture label prediction, and iterative patch sampling for feature-guided inpainting.
- **Extensive effectiveness validation:** Experiments demonstrate that our proposed PRISM method is not only effective for dual-color infrared detector DPCs but also achieves good performance on four public multimodal datasets, preliminarily validating the method’s modality extensibility.

## 2. Related Work

We organize prior work along three complementary themes—(i) defective pixel correction, (ii) exemplar-based image inpainting, and (iii) deep-learning inpainting—and then summarize in Table 1 how PRISM differs from representative methods across input modality, structural guidance, texture guidance, training requirement, and cross-band information usage.

### 2.1. Defective Pixel Correction Methods

**Single-band Correction Techniques:** Traditional defective pixel correction techniques typically exploit redundancy in the spatial, temporal, or spectral domains. Early single-frame algorithms, such as neighborhood mean replacement [9] and Gaussian filtering [10], offer low computational complexity but are limited to isolated discrete defects. Improved methods incorporating edge-awareness and adaptive gradient thresholds [11,12] can better preserve edges and high-frequency textures; however, they still struggle with large-area continuous missing regions due to insufficient local reference information. Scene-based methods [13,14,15] introduce temporal redundancy through frame-to-frame motion estimation and multi-frame fusion. While effective for small clusters, these approaches heavily depend on relative motion between the detector and scene, leading to severe performance degradation in static scenes or when motion-induced registration errors are large.

**Dual-band Spectral Correction:** For severe static continuous defects, cross-band spectral redundancy has proven to be a more robust auxiliary signal source. Nguyen et al. [18] pioneered this direction by proposing the dual-band statistical lookup and dual-band image painting methods, which leverage pixel-wise correlations between LWIR and MWIR bands. These methods achieved promising results on similar bands dominated by thermal emission. However, DSL and DIP rely on the assumption of direct pixel-wise intensity mapping between bands. This assumption breaks down in SWIR/MWIR systems where the two bands capture fundamentally different physical signals—SWIR primarily measures reflected solar radiation, while MWIR captures thermal self-emission. Consequently, direct intensity mapping can introduce modality-specific artifacts (e.g., reflective features incorrectly propagated to thermal regions), motivating the need for feature-level rather than pixel-level cross-band information transfer.

### 2.2. Exemplar-Based Image Inpainting

Exemplar-based inpainting methods synthesize missing content by copying patches from known regions. The seminal work of Criminisi et al. [16] introduced a priority-driven greedy filling strategy based on confidence and data terms. To avoid local optima, subsequent works formulated inpainting as a global optimization problem [19,20] albeit at increased computational cost. The PatchMatch algorithm [21] dramatically accelerated patch search through random propagation, becoming a cornerstone of modern exemplar-based methods.

Recognizing that pure texture synthesis often fails to recover broken geometric structures, researchers introduced explicit structure propagation mechanisms. Sun et al. [22] proposed completing structural curves first via tensor voting before texture synthesis. Liu et al. [23] and Wu et al. [24] developed edge-extension engines to maintain geometric boundary continuity. Most relevant to our work, Huang et al. [17] exploited planar structure regularity (e.g., translational symmetry) to guide patch synthesis, achieving significant improvements on architectural scenes.

Beyond planar regularity, hierarchical and multi-scale restoration strategies have been introduced to better preserve structural and textural coherence across scales. Representative traditional frameworks established the coarse-to-fine paradigm: Hertzmann et al. [36] introduced image analogies with multi-scale texture transfer, Drori et al. [37] proposed a fragment-based completion that refines structure from coarse to fine, Wexler et al. [19] formulated inpainting as a global space–time optimization, and Pritch et al. [38] developed shift-map editing for label-consistent completion. Wang et al. [39] proposed a pyramid-model-based down-sampling scheme that propagates structure guidance from coarse to fine levels to accelerate inpainting, while Ma et al. [40] presented an efficient image/video inpainting framework built upon a coarse-to-fine hierarchy. These structure-aware, multi-scale designs motivate the hierarchical feature decomposition employed in our CMFA module, which likewise reconstructs structural skeletons before refining texture details.

While these structure-guided methods excel in single-band RGB images, they do not account for the unique challenges of cross-band feature alignment in infrared imagery, where correspondence may be disrupted by modality-specific features.

### 2.3. Deep Learning Approaches for Image Inpainting

Deep learning has advanced image inpainting through large-scale data-driven representation learning. Early encoder–decoder architectures [25,26] adopted a two-stage pipeline combining edge map prediction with edge-guided texture synthesis. Attention mechanisms [27,28,29] were subsequently integrated to enhance global context modeling. More recently, Transformer-based methods [34,35] have demonstrated improved capability in capturing long-range dependencies, while efficiency-focused designs such as PEIPNet [31] attempt to reduce computational overhead through depthwise separable convolutions.

When applied to dual-band infrared DPC correction, deep learning methods face inherent limitations. Data-driven generation may produce structures inconsistent with the radiometric properties of the target band [32]. Single-band input cannot exploit complementary information from the reference band, which is essential for resolving ambiguities in severely corrupted regions. Furthermore, large model sizes and computational demands pose challenges for deployment on resource-constrained infrared imaging platforms. Recent work in depth inpainting [33,41] has shown that cross-modal structural guidance remains effective even when imaging characteristics differ between modalities, motivating our CMFA module design. More recently, multimodal fusion and inpainting have been advanced by Transformer-based and diffusion-based approaches. Cross-modal Transformer architectures [42] have demonstrated improved long-range dependency modeling for infrared–visible image fusion. Diffusion models such as Dif-Fusion [43] generate multi-channel data distributions to enhance multi-source information aggregation. For inpainting itself, uncertainty-aware adaptive feedback networks [44] have been proposed to handle large holes where image guidance is insufficient. However, these methods are primarily designed for RGB-level multimodal fusion or generic image inpainting; they do not address the specific challenge of cross-band physical consistency (reflection vs. thermal emission) that is central to dual-band infrared DPC correction nor do they operate in a training-free regime suitable for resource-constrained infrared imaging platforms. To clarify how PRISM differs from prior defective-pixel correction and image inpainting methods, Table 1 summarizes their characteristics along five dimensions: input modality, structural guidance, texture guidance, training requirement, and information usage.

PRISM simultaneously achieves cross-band information utilization, multi-level structure awareness, and training-free operation for dual-band infrared DPC correction.

## 3. Proposed Method

**Figure 2:**
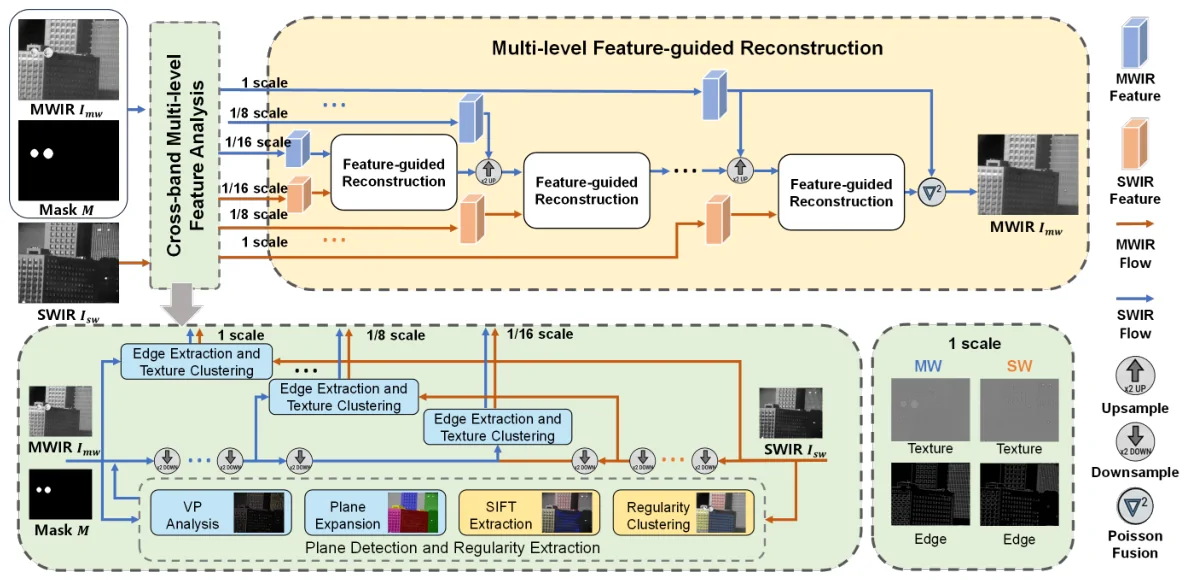
Framework of the proposed dual-band defective pixel correction method. The pipeline consists of two stages: (1) Cross-band Multi-level Feature Analysis (CMFA), which takes the dual-band inputs $I_{sw}$ and $I_{mw}$ together with the defect mask M and extracts three levels of cross-band priors—spatial layout (plane membership and regularity vectors), structural edges, and texture labels; (2) Multi-level Feature-Guided Reconstruction (MFGR), which uses these priors together with the original dual-band images to restore the MWIR image in a coarse-to-fine multi-scale pyramid.

Figure 2 illustrates the overall pipeline of PRISM, which consists of two main stages: Cross-band Multi-level Feature Analysis and Multi-level Feature-Guided Reconstruction. CMFA aims to leverage physical correlations between SWIR and MWIR bands, along with the pixel-wise registration property of dual-band detectors, to provide more accurate guidance for the correction process at three levels: spatial relationships, structural skeletons, and texture details. MFGR utilizes the multi-level feature analysis results to recover corrupted images through multi-scale iterative reconstruction.

Specifically, to correct large-area DPCs, CMFA first decomposes the scene into a hierarchical representation consisting of spatial layout, structural edges, and texture patterns. Subsequently, MFGR reconstructs the missing structural skeleton in a hierarchical manner and assigns texture labels to missing pixels. Based on the predicted structural framework and texture labels, MFGR performs sampling and iteration within specified regions, progressively upsampling to coarser scales until achieving iterative image restoration from low to high resolution.

Throughout this paper, we denote by Ω the target region of defective pixels to be inpainted, $\bar{\Omega }$ the source region of known valid pixels, and ∂Ω the boundary contour. $I_{sw}$ and $I_{mw}$ represent the SWIR and MWIR band images, respectively. $E_{sw}$ and $E_{mw}$ are their corresponding binary edge maps. $L_{T}$ denotes the texture label map obtained from clustering. $P_{k}\left(x\right)$ refers to an image patch centered at position x in band *k*, and M(p) is a binary mask indicating known (1) or missing (0) pixels.

### 3.1. Cross-Band Multi-Level Feature Analysis

The core challenge in cross-modal feature extraction lies in the significant photometric differences between dual bands (SWIR reflection vs. MWIR thermal emission). These physical mechanism differences often lead to local contrast inversion and modality-specific feature generation. Traditional feature analysis methods are extremely sensitive to absolute brightness and gradient direction, making robust information interaction difficult across bands.

To address this issue, we propose CMFA, which is a cross-modal multi-level feature decomposition method that can selectively retain reliable correction cues while suppressing inconsistent cross-spectral differences. Specifically, for the fine-grained repair of dual-band DPC regions, CMFA extracts cross-band information through three synergistic dimensions:

- **Spatial Layer:** dedicated to reconstructing macroscopic scene information and global regularity patterns.
- **Edge Layer:** ensures adaptive alignment and effective inward expansion of local high-frequency scene structures.
- **Texture Layer:** locks local material attributes through joint spectral clustering, fundamentally suppressing feature confusion and error diffusion between heterogeneous pixels.

Notably, for edge and texture features, we construct image pyramids and perform joint extraction in multi-scale space.

#### 3.1.1. Plane Detection and Regularity Extraction (Spatial Layer)

High-level spatial priors, particularly planar geometry and global regularity, are crucial for constraining the topological reconstruction of large-scale defective pixel clusters. In urban and road scenes, such high-level priors have been proven effective in avoiding structural distortion during inpainting [17]. In our implementation, we adopt this framework as the basis of our spatial prior extraction.

However, the target band (MWIR) suffers not only from DPC destruction but also geometric edge blurring caused by its thermal radiation imaging mechanism. In contrast, the reference band (SWIR) relies on reflection-based imaging, offering higher geometric fidelity. Therefore, we propose a spatial prior extraction mechanism that leverages the highly reliable physical geometric information from the reference band to strongly guide planar and periodicity inference for missing regions in the target band.

We first extract vanishing points (VPs) and their corresponding parallel 3D line segment clusters from the SWIR image. To convert discrete line segments into continuous probability distributions, we apply wide Gaussian-kernel spatial diffusion filters to VP lines, generating VP support density maps. Subsequently, by computing joint products of orthogonal VP density maps belonging to the same plane, we obtain the joint spatial support response for each plane. After normalization, so that the membership probabilities of all candidate planes (plus an additional fronto-parallel plane with a small uniform density) sum to one at each pixel, we acquire probabilistic information regarding pixel membership to different planes. This planar probability is transferred as cross-modal prior information into the DPC region of MWIR.

Since repetitive structures in man-made scenes are typically equidistantly distributed in 3D physical space but undergo distortion when imaged onto 2D by the detection system, we extract accurate periodicity features by mapping matched feature points via planar homography into a rectified canonical 2D space. In this space, genuine physical periodic translations manifest as spatial-invariant displacement vectors that form high-density clusters, which we detect via mean-shift clustering (bandwidth of 10 pixels; modes with fewer than 10 members are rejected) with parameter settings following [17]. After extracting Scale-Invariant Feature Transform (SIFT) features [45] from both bands, we design a plane-constrained matching strategy using Kd-tree nearest neighbor search (distance threshold set to 0.15). For any candidate plane $\Pi_{m}$, we only retain feature pairs satisfying high posterior probability constraints, i.e., requiring the joint planar membership probability of matched pairs $\left(x_{1},x_{2}\right)$ to exceed a threshold $\tau_{p}=0.5$, following [17].

The transformation matrix mapping pixels from image space to each plane’s $\Pi_{m}$ canonical space is denoted as the planar homography matrix $H_{m}$, which will be used in subsequent patch sampling stages for cross-planar distortion correction. The planar constraint serves dual purposes: (i) guiding the patch sampling process to encourage iterative sampling along planar directions; (ii) reducing distortion errors when sampling patches from different planes. Meanwhile, the regularity constraint will be explicitly integrated into the subsequent global objective function (see $E_{\mathrm{reg}}$ term in Equation (8)).

#### 3.1.2. Adaptive Edge Extraction (Edge Layer)

The physical imaging mechanism differences between SWIR and MWIR bands make cross-modal edge alignment highly challenging. Traditional edge detectors tend to fail when facing significant dynamic range differences. To achieve multi-scale adaptive edge extraction within the PRISM framework while protecting non-shared thermal features and providing structural guidance for subsequent DPC region repair, we propose an adaptive edge extraction strategy based on cross-modal consistency.

This strategy accomplishes two goals: (i) identifying strong edges for cross-band alignment; and (ii) mining weak edges around missing regions to assist structure reconstruction.

First, to reduce interference from manual global threshold settings, we employ the classical Canny edge detection algorithm [46] to extract the SWIR edge map $E_{sw}$. On this basis, we formulate the search for the optimal MWIR edge extraction threshold $\tau^{*}$ as a cross-modal structure fidelity maximization problem. Considering that MWIR contains unique thermal target edges (i.e., regions that should not be forced into alignment with SWIR), we introduce an adaptive weight $\omega_{i}$ with cross-band thermal saliency awareness into the objective function. The optimal threshold $\tau^{*}$ is obtained by maximizing the following modified Pratt’s Figure of Merit (FOM) [47] function F:

(1) $$F\left(\tau \right)=\frac{1}{\max(N_{mw}\left(\tau \right),\;N_{sw})}\sum_{i=1}^{N_{mw}\left(\tau \right)}\left[\left(1-\omega_{i}\right)\frac{1}{1+\alpha d_{i}^{2}}+\beta \omega_{i}\right],$$

where $N_{mw}\left(\tau \right)=\left|E_{mw}\left(\tau \right)\right|$ and $N_{sw}=\left|E_{sw}\right|$ represent the number of MWIR edge pixels at current threshold τ and baseline edge pixels, respectively. The denominator uses their maximum value, effectively penalizing massive edge loss caused by extremely high thresholds (avoiding trivial solutions). The distance term $d_{i}=\min_{p\in E_{sw}}{∥x_{i}-p∥}_{2}$ denotes the minimum Euclidean distance between the *i*-th MWIR edge pixel $x_{i}\in E_{mw}\left(\tau \right)$ and the reference set $E_{sw}$. The parameter α (set to 1/9) is a constant factor controlling the spatial displacement penalty scale.

To accurately preserve unique edges, the weight $\omega_{i}\in \left[0,1\right]$ is computed from cross-band local gradient energy contrast: when pixel $x_{i}$ exhibits a strong gradient in MWIR but weak gradient in the corresponding SWIR region, it is identified as a unique thermal edge and $\omega_{i}\to 1$. In this case, the term is exempt from spatial distance penalty while being preserved through constant factor β (set to 0.5). Conversely, when the edge belongs to cross-band shared structures, $\omega_{i}\to 0$, and the objective strongly constrains its spatial alignment with $E_{sw}$.

This mechanism enables PRISM to precisely extract cross-band strong edges and main structures while robustly preserving genuine thermal radiation representations beyond visual clutter.

Due to inter-band radiance differences and detector physical defects (such as DPCs), relying solely on strong edges cannot provide continuous structural constraints. Therefore, we perform secondary mining for key structural clues in missing regions and their neighborhoods. Utilizing positions of edge line segments spanning normal regions and missing regions from the prior edge map $E_{sw}$, we locally apply gradient enhancement on the MWIR gradient map combined with non-maximum suppression (NMS) for directed edge searching. By computing consistency between newly extracted structural lines and prior structures in terms of spatial coherence and gradient direction, we determine whether these weak edges are valid extensions of effective structures.

Notably, weak edge extraction is bidirectional: the target band mines reference band edge information in reverse, finally combining strong and weak edges. This targeted weak edge mining for missing regions provides high-fidelity prior cues for the subsequent decoupled repair of structure and texture regions.

#### 3.1.3. Spectral Texture Clustering (Texture Layer)

Existing image inpainting methods are prone to cross-modal error propagation, which is primarily caused by the confusion of pixel features associated with wavelength-dependent different materials. We propose a joint spectral energy feature-based texture clustering method that classifies local texture features of known regions at the pixel level, assigning labels representing material attribute differences. We perform classification at different pyramid levels to extract feature information with varying receptive fields.

We decompose material features into two components: intrinsic radiation intensity and local texture intensity. SWIR imaging exhibits significant textural characteristics while MWIR imaging emphasizes intrinsic radiation. To characterize inter-band variations of different materials, we combine texture intensity and radiation intensity into a joint feature vector representing local material properties, defined as $E_{k}\left(x,y\right)={\left[A_{k}\left(x,y\right),T_{k}\left(x,y\right)\right]}^{T}$, where $A_{k}\left(x,y\right)$ and $T_{k}\left(x,y\right)$ represent material radiation characterization and texture intensity for band k∈{sw,mw}, respectively.

To ensure computational efficiency, we adopt Relative Total Variation [48] to separate low-frequency radiation from high-frequency textures for assessing intrinsic radiation intensity and texture variation degrees at different spatial positions. Each pixel is then mapped to a cross-band joint spectral feature vector $e\left(x,y\right)=[E_{mw}\left(x,y\right),E_{sw}\left(x,y\right)]$. To adaptively capture complex material attributes without pre-specifying cluster count, we perform MeanShift clustering [49] on the feature vector e(x,y). By iteratively moving data points toward local density maxima (modes) $\mu_{l}$ in the joint feature space, the scene is partitioned into a texture label map $L_{T}$:

(2) $$L_{T}\left(x,y\right)=l\Leftrightarrow e_{i}^{\left(\infty \right)}=\mu_{l},\;l\in \left\{1,\ldots ,N_{l}\right\},$$

where $e_{i}^{\left(\infty \right)}$ denotes the convergence point of e(x,y), and $N_{l}$ is the adaptively determined number of texture categories.

Notably, the derived intra-cluster statistics (mode $\mu_{l}$ and texture covariance $\Sigma_{l}$) serve as explicit prior constraints in the reconstruction stage. They provide reference distributions for estimating posterior probabilities of missing pixels, enabling a description of target missing pixels’ texture performance from a probabilistic perspective based on reference band pixel features.

### 3.2. Multi-Level Feature-Guided Reconstruction

We propose MFGR, which is a patch-based algorithm to reconstruct missing pixels within DPC region Ω. We formulate this stage as a multi-scale cascaded optimization process, where each level contains three sub-steps: *structure propagation, texture relabeling,* and *patch sampling with iteration*. The overall repair follows a coarse-to-fine iterative strategy: starting from the coarsest scale to construct a basic topological skeleton and texture labels for missing regions, progressively upsampling and embedding repaired structures into larger scales as prior guidance, combining with newly emerging refined structural edges for local refinement, until restoration to original resolution.

#### 3.2.1. Structure Propagation with Adaptive Dual-Band Priors

Our proposed dual-band prior-based structure restorer aims to use the reference band’s structure map $E_{sw}$ as guidance for repairing target band structure $E_{mw}$. To balance computational efficiency with accurate fine structure recovery, we embed the entire structure restorer within a multi-scale framework ranging from structural skeleton to structural details.

Specifically, structure repair begins at the image’s minimum scale. At this scale, the repair process rapidly constructs a basic topological skeleton for missing regions with minimal computational overhead. After preliminary repair, the algorithm upsamples this basic structure to larger scales and directly embeds it into missing regions at that scale as prior guidance. Subsequently, the algorithm re-evaluates the reference band $I_{sw}$: if resolution improvement reveals edge pixels not present at the previous scale (determined by performing per-pixel differencing on adjacent scale edge maps, where pixels with a differential response exceeding threshold $\epsilon_{e}$ are judged as new features), the restorer is re-activated for local refinement targeting these new features; if the scale-to-scale differential response falls below $\epsilon_{e}$, no new features are detected and processing continues to the next coarser scale. This “upsample–structure embed–new edge refine” process iterates layer-by-layer until restoration to the original maximum resolution scale.

Within any specific scale level, physical mechanism differences between SWIR reflection and MWIR thermal radiation may lead to significant structural inconsistencies between bands (e.g., non-common edges and spatial offsets of related thermal boundaries). Addressing these differences, each layer’s structure restorer calibrates structurally discrepant input sketches into precise structure guidance maps with core execution flow as follows:

First, define anchor points as intersections between structural curves and blind zone boundary ∂Ω. Valid structures within missing region Ω must be anchored on confirmed anchor points from target band image $I_{mw}$. Furthermore, leveraging detector operating characteristics in man-made scenes, we utilize the inherent spatial regularity extracted in the Spatial Layer section (e.g., repetitive architectural textures). By identifying global repetition patterns and structural translation periods within known valid regions, we extract reliable structure anchors $a_{val}$ and infer virtual anchors $a_{vir}$ along periodic directions inside Ω. This mechanism effectively transfers known periodic structures and topological constraints into DPCs, greatly compensating for the reference scarcity caused by cross-modal differences by enforcing intra-band periodic consistency, ensuring the completeness of the predicted structural grids.

Second, we uniformly sample on known structural edges within Ω’s neighborhood N(Ω) (i.e., known pixel band outside ∂Ω), constructing equal-sized point sets $P_{mw}=\left\{p_{1},\ldots ,p_{N}\right\}\subset E_{mw}$ and $P_{sw}=\left\{q_{1},\ldots ,q_{N}\right\}\subset E_{sw}$ from $E_{mw}$ and $E_{sw}$, respectively. At current scale, we compute cross-correlation between reference point set $P_{sw}$ and known edge point set $P_{mw}$: for N=1 pixel-wise alignment, directly use the $L_{1}$ distance to measure single-point correlation; for N>1 region-level alignment, compute the correlation between the local area means centered at each sampling point. Addressing the potential overall slight spatial offsets at certain edges due to spectral differences, we compute the optimal displacement vector $d^{*}$ between two edge point sets to minimize the mean squared error (MSE) between band structure point sets. This formulation follows the standard least-squares alignment principle widely used in feature matching [45], where the optimal offset is obtained by minimizing $\sum_{i=1}^{N}{∥p_{i}-\left(q_{i}+d\right)∥}_{2}^{2}$ over d, yielding the closed-form mean displacement between the two point sets.

**Figure 3:**
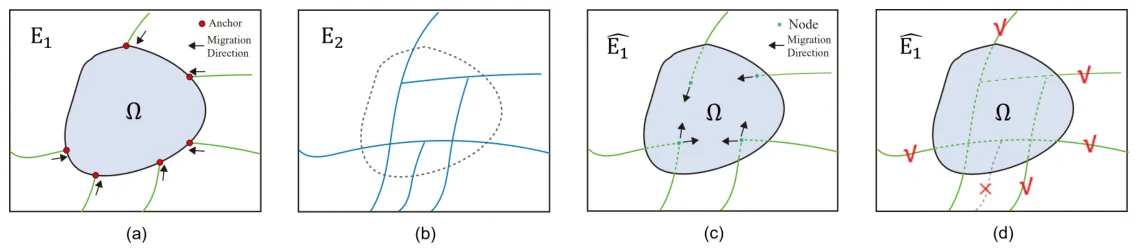
Schematic diagram of the structure propagation mechanism using adaptive dual-band priors. (**a**) Initial structure in target band $C_{mw}$, where red points indicate anchors extending toward missing region Ω. (**b**) Structure priors from reference band $C_{sw}$. (**c**) Intermediate structure transfer process, introducing nodes from $C_{sw}$ to guide propagation direction. (**d**) Final recovered topology, where gray dashed lines with crosses indicate unreliable structural candidates removed via adaptive reliability verification.

Subsequently, we apply this global offset $d^{*}$ to transform candidate reference structures $C_{sw}$ (i.e., candidate sets of SWIR structure curves crossing Ω after offset correction) and associated cross-anchors (i.e., anchors mapped from $P_{sw}$ via $d^{*}$ transformation into the MWIR coordinate system). This process ensures the completed structure framework aligns topologically with actual geometric positions in the target band. As shown in Figure 3, discrete clues that fail to anchor or match are classified as redundant artifacts and removed from the propagation graph.

Finally, utilizing the refined structure prior and inferred junctions and anchors, the algorithm performs path planning for connections between sampled edge points. The restorer conducts confidence assessment based on directional correlation among edge point sets and topological reliability among anchors, prioritizing the transmission of high-confidence structural clues through iterative propagation. This confidence-weighted propagation scheme is similar in spirit to the structure propagation framework of Sun et al. [22], where structural clues are prioritized based on a confidence score. Following this principle, we define the propagation confidence *C* as a joint exponential decay function of directional similarity and spatial distance: $C\left(p_{i},p_{j}\right)=\exp\left(-\alpha_{c}\;\cdot \;\Delta \theta_{ij}\right)\;\cdot \;\exp(-\beta_{c}\;\cdot \;∥p_{i}-p_{j}{∥}_{2})$, where $\Delta \theta_{ij}$ denotes the tangent direction angle difference at two edge points, and $\alpha_{c}$, $\beta_{c}$ are constants controlling directional and spatial decay rates. High-confidence (*C* value large) structural clues serve as reliable skeletons prioritized for propagation. This step completes fine-grained repair at the current scale, outputting the processed complete structure as a solid foundation for upsampling and propagation to the next larger scale.

#### 3.2.2. Texture Relabel with Adaptive Dual-Band Priors

**Figure 4:**
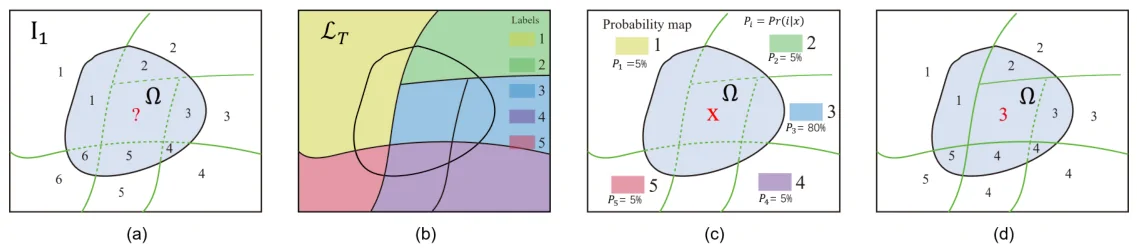
Adaptive texture label expansion via dual-band priors. (**a**) Preliminary label map with unlabeled holes. (**b**) SWIR label map M from CMFA. (**c**) Posterior $p(l_{mw}|p)$ under dual-band guidance. (**d**) Final MAP-expanded labels.

The classic “Texture by Numbers” approach [36] effectively addresses the inherent cross-blurring problem in traditional texture synthesis. By leveraging structure curves from the preceding propagation step to partition the blind region Ω into sub-regions, texture labels can be smoothly extended along structural boundaries, as shown in Figure 4a. However, isolated holes $\Omega_{h}$ completely surrounded by blind pixels admit no such extension.

We resolve this through a Bayesian inference framework grounded in cross-band statistical dependencies learned from CMFA. The posterior computation relies on standard multivariate Gaussian conditional inference theory [50]; we summarize the key formulas below. Recall from Section 3.1.3 that clustering the joint features over the valid region $\bar{\Omega }$ yields physically meaningful material categories spanning both bands, which are parameterized by joint Gaussian statistics (mean $\mu_{l}$ and covariance $\Sigma_{l}$).

For any pixel $p\in \Omega_{h}$, only its SWIR feature $e_{sw}\left(p\right)$ is observable. We first evaluate the probability of each texture class based solely on this available data to obtain the SWIR-domain class posterior:

(3) $$p\left(l_{sw}|p\right)=\frac{\pi_{l_{sw}}\;N\left(e_{sw}\left(p\right);\mu_{l_{sw},sw},\Sigma_{l_{sw},sw,sw}\right)}{\sum_{k=1}^{L}\pi_{k}\;N\left(e_{sw}\left(p\right);\mu_{k,sw},\Sigma_{k,sw,sw}\right)},$$

where $\pi_{l_{sw}}$ is the empirical class prior derived from the valid region, *k* is the summation dummy variable, and N is the Gaussian probability density function.

To map this SWIR-indexed distribution onto the target MWIR label space, we leverage the joint distribution. Because the two bands are statistically correlated, observing the SWIR feature alters our expectation of the missing MWIR feature. Using conditional Gaussian inference, we can compute the statistically optimal MWIR prediction—the conditional expectation ${\tilde{\mu }}_{l_{mw}|sw}\left(e_{sw}\right)$—given the SWIR evidence.

We then define a cross-band transition weight $T(l_{mw}\leftarrow l_{sw};\;p)$ that measures the compatibility between a source SWIR class $l_{sw}$ and a target MWIR class $l_{mw}$. This weight is formulated by comparing the distance between their respective MWIR predictions, effectively quantifying cross-band label consistency. Finally, marginalizing over all possible SWIR assignments yields the final MWIR label posterior:

(4) $$p\left(l_{mw}|p\right)=\sum_{l_{sw}=1}^{L}T\left(l_{mw}\leftarrow l_{sw};\;p\right)\;p\left(l_{sw}|p\right).$$

This posterior is fully grounded in the observable SWIR evidence while strictly respecting the cross-band statistics. In the subsequent texture optimization, it serves as an uncertainty-aware soft constraint for filling $\Omega_{h}$ (see Equation (9)), preserving cross-band structural consistency during patch sampling.

#### 3.2.3. Reconstruction Objective Function

We propose a specialized objective function for dual-band reconstruction that aims to find the optimal source patch offset $q\in \bar{\Omega }$ for each target pixel p∈Ω based on known prior information. Our formulation extends the global optimization framework of Wexler et al. [19] and the exemplar-based inpainting paradigm [16] with the patch search accelerated by the PatchMatch algorithm [21]. The objective function is formulated as follows:

(5) $$\begin{array}{cc} \; & \min_{\left\{q\right\}}\sum_{p\in \Omega }\left(E_{\mathrm{app}}\left(p,q\right)+E_{\mathrm{coh}}\left(p,q,m_{p}\right)\right) \end{array}$$

(6) $$\begin{array}{cc} \; & E_{\mathrm{app}}\left(p,q\right)=\left(1-\lambda_{\mathrm{app}}\right){∥M\left(p\right)⊙(P_{mw}\left(p\right)-P_{mw}\left(q\right))∥}_{2}+\lambda_{\mathrm{app}}{∥P_{sw}\left(p\right)-P_{sw}\left(q\right)∥}_{2} \end{array}$$

(7) $$\begin{array}{cc} \; & E_{\mathrm{coh}}\left(p,q,m_{p}\right)=\lambda_{\mathrm{reg}}E_{\mathrm{reg}}+\lambda_{\mathrm{tex}}E_{\mathrm{tex}}+\lambda_{\mathrm{prox}}E_{\mathrm{prox}}+\lambda_{\mathrm{nbr}}E_{\mathrm{nbr}}, \end{array}$$

Here, $m_{p}$ encodes periodic regularity vectors and texture labels, and ${∥\;\cdot \;∥}_{2}$ denotes the $L_{2}$ distance. For the appearance cost $E_{\mathrm{app}}$, $P_{k}\left(x\right)$ represents a patch centered at position x in band *k*, M(p) is a binary mask (1 for known pixels, 0 for missing pixels), and ⊙ denotes element-wise multiplication, ensuring that target band appearance matching is computed only on known overlapping regions. The parameter $\lambda_{\mathrm{app}}$ (set to 0.5) modulates the reference band’s contribution to leverage reliable cross-band structural cues for synthesis guidance. The coherence cost $E_{\mathrm{coh}}$ imposes higher-level semantic constraints through regularity ($E_{\mathrm{reg}}$), texture ($E_{\mathrm{tex}}$), and proximity ($E_{\mathrm{prox}}$) terms with weight parameters set as $\lambda_{\mathrm{reg}}=10$, $\lambda_{\mathrm{tex}}=20$, $\lambda_{\mathrm{prox}}=1$, and $\lambda_{\mathrm{nbr}}=5$. Each coherence cost term is detailed below:

- **Regularity Cost ($E_{\mathrm{reg}}$):** This term utilizes the structural periodicity identified in CMFA to encourage sampling source patch q from positions predicted by the inherent lattice periodicity relative to p:
  (8) $$E_{\mathrm{reg}}\left(p,q,m_{p}\right)=\frac{∥T\left(p,k_{u},k_{v}\right){-q∥}_{2}}{0.5\sqrt{k_{u}^{2}+k_{v}^{2}}},$$
  where T represents coordinates transformed based on the periodic vector $\left(k_{u},k_{v}\right)$.
- **Texture Cost ($E_{\mathrm{tex}}$):** We incorporate the derived posterior probability prior as a negative log-likelihood penalty term:
  (9) $$E_{\mathrm{tex}}\left(p,q,m_{p}\right)=-\log\left(Pr\left(l_{q}|p\right)\right)-\log\left(Pr\left(l_{q}|q\right)\right),$$
  where $l_{q}$ is the mw texture label of q. This cost penalizes retrieving patches from different texture categories, ensuring synthesized content respects texture labels. This loss applies equally to edge pixels with identical constraint effects.
- **Proximity Cost ($E_{\mathrm{prox}}$):** Motivated by the spatial proximity penalty of Huang et al. [17], which improves synthesis by favoring nearby valid source patches over distant or differently scaled regions, we adapt their formulation and redesign the following proximity term for inpainting in our method. The proximity loss is defined as
  (10) $$E_{\mathrm{prox}}\left(p,q\right)=\frac{{∥p-q∥}_{2}^{2}}{d{\left(p\right)}^{2}+\gamma^{2}},$$
  where p and q denote spatial coordinates of the target patch and matched source patch, respectively. The $d{\left(p\right)}^{2}$ term represents the squared distance from target position p to the nearest boundary of known (unoccluded) regions. The parameter $\gamma^{2}$ controls the strength of this proximity penalty; we empirically set γ=M/8, where *M* denotes the maximum spatial dimension of the image.
- **Neighborhood Cost ($E_{\mathrm{nbr}}$):** To maintain texture continuity, neighboring pixels should sample from coherent source regions. Motivated by Newson et al. [51], who showed that penalizing the squared sum of offset differences between neighboring NNFs is an effective constraint, we build on this principle and design a new neighborhood constraint that replaces their quadratic penalty with a robust exponential kernel to suppress outlier offsets:
  (11) $$E_{\mathrm{nbr}}\left(p,q\right)=\frac{1}{\left|N(p)\right|}\sum_{i=1}^{\left|N(p)\right|}\left[1-\exp\left(-\frac{∥\delta_{i}\left(q\right)-\delta_{i}{\left(p\right)∥}_{2}^{2}}{2\sigma^{2}}\right)\right],$$
  where N(p) denotes the set of previously compensated neighbors of p, and |N(p)| is the number of these valid neighbors. Let $p_{i}\in N\left(p\right)$ be the *i*-th neighbor and $q_{i}$ its corresponding sampling coordinate in the source image. Here, $\delta_{i}\left(p\right)=p-p_{i}$ represents the relative spatial offset in the target region, while $\delta_{i}\left(q\right)=q-q_{i}$ is the corresponding relative offset in the source region. This robust kernel suppresses mapping outlier influence and prevents over-weighting, ensuring the stability of the local optimization process.

#### 3.2.4. Pixel Patch Sampling and Iterative Propagation Strategy

**Figure 5:**
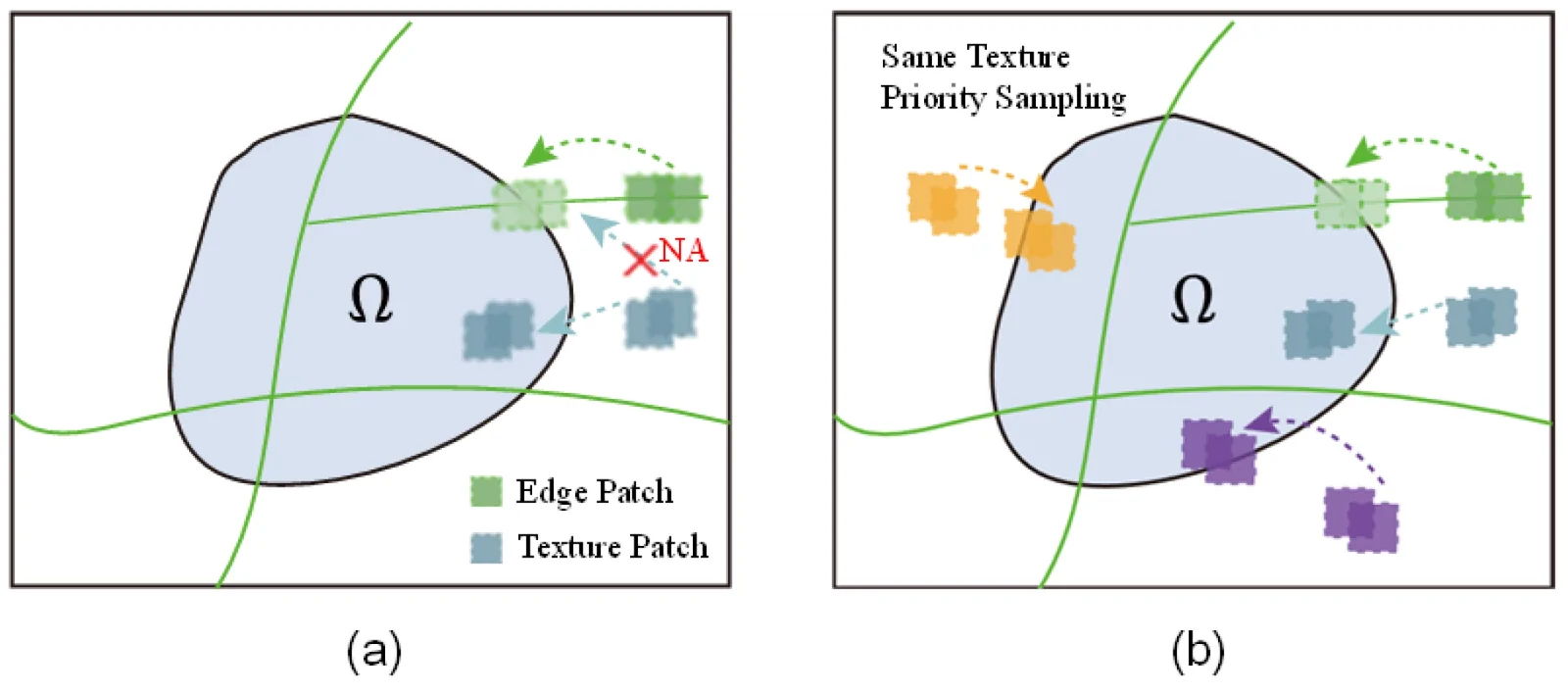
Schematic diagram of pixel patch sampling and iterative propagation strategy. (**a**) **Semantically Constrained Propagation:** The search space for structure patches (green) is strictly limited to identified skeleton positions, while texture patches (blue) are constrained to propagate within regions sharing the same semantic label, avoiding cross-category matching errors. (**b**) **Posterior-Guided Sampling Strategy:** Illustration of same-texture-priority sampling mechanism, which biases the search space toward regions sharing identical material attributes to improve sampling efficiency.

We enhance the standard PatchMatch algorithm [21] by deeply integrating the plane distribution and texture regularity extracted in Section 3.1.3. In our implementation, during the search and propagation stages, we perform multi-scale iterative reconstruction starting from the coarsest scale and progressively refining toward the original resolution. To prevent error propagation across scales, we employ two sampling strategies to constrain the search space, along with cross-planar homographic rectification to eliminate inter-plane distortion, as illustrated in Figure 5 and detailed as follows:

- Semantically Constrained Propagation: To reduce matching errors while preserving structure pixel saliency, we enforce category-specific constraints beyond the loss function. Specifically, the search space for structure patches is strictly confined to identified structure skeleton positions, while texture patches are constrained to iteratively search and propagate within regions having identical semantic labels, avoiding cross-category matching errors.
- Posterior-Guided Sampling: For the candidate sampling of texture patches, we first sample the texture index *l* for target pixel p from the posterior distribution Pr(l|p); then, we randomly draw candidate source locations q from the set of pixels with texture label *l* in known regions. This strategy effectively biases the search space toward regions statistically belonging to the same material category, significantly improving sampling efficiency compared to uniform random search.

Furthermore, to address scenarios where target patch $P_{k}\left(p\right)$ and source patch $P_{k}\left(q\right)$ reside on different geometric planes causing distortion, we introduce Cross-Planar Homographic Rectification. Utilizing the plane homography matrices $H_{m}$ extracted in the Spatial Layer section, before computing matching losses, we spatially transform the source patch $P_{k}\left(q\right)$ and its neighborhood into the parameter space of target plane $\Pi_{m}$. This homographic rectification mitigates affine deformation effects, ensuring accurate texture synthesis even when crossing planes with different orientations.

Regarding computational complexity, MFGR’s primary overhead concentrates on multi-scale iterative patch matching and path planning. For the PatchMatch step, instead of a brute-force $O(|\Omega |\;\cdot \;|\bar{\Omega }|)$, the randomized search reduces the complexity to $O(|\Omega |\;\cdot \;K_{pm}\;\cdot \;S)$ per scale, where $K_{pm}$ is the iteration count and *S* is the patch area. Because an image pyramid is employed, the pixel count decays exponentially across *L* levels. Consequently, the geometric series of operations converges, and the total matching runtime is strongly bounded and dominated by the highest-resolution scale. Overall, the time complexity can be approximated as $O(|\Omega |\;\cdot \;K_{pm}\;\cdot \;S+C_{\mathrm{path}})$, where $C_{\mathrm{path}}$ represents the path-planning overhead.

## 4. Experimental Evaluations

This section evaluates PRISM against dual-band blind pixel cluster correction algorithms DSL and DIP [18], the classical image inpainting algorithm by Huang et al. [17], as well as state-of-the-art deep learning-based image inpainting algorithms PEIPNet [31] and ZITS++ [35]. Furthermore, we design ablation experiments to validate the effectiveness of prior information extraction modules, testing configurations that progressively remove all prior guidance (degrading to a baseline algorithm) and incrementally adding different guidance information, thereby demonstrating the impact of more precise multi-level feature priors on image inpainting performance.

### 4.1. Experimental Setup

**Figure 6:**
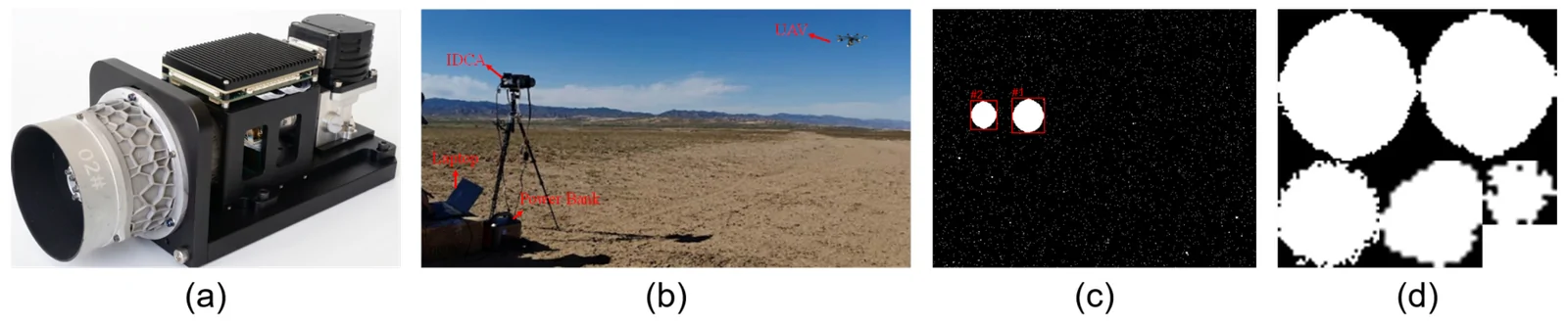
(**a**) Integrated detection system of the dual-band SW/MW T2SL FPA; (**b**) field deployment for data acquisition; (**c**) blind-pixel mask of detector #H317; (**d**) DPCs used during evaluations.

**Datasets:** We collected real dual-band detector defect data and image sequences for evaluation using multiple self-developed dual-band detector systems. Figure 6a illustrates the data acquisition setup. The experimental apparatus, shown in Figure 6b, centers on a T2SL dual-band detector prototype integrated with a Stirling Integrated Detector Dewar Cooler Assembly (IDDCA). The optical front-end employs a coaxial reflective optical system with an effective bandwidth range of 1–6 μm. The sensor operates at a Stirling cooling temperature of 87 K with the MW band’s Noise Equivalent Temperature Difference (NEDT) below 30 mK and the SW band below 60 mK. This dataset encompasses diverse environmental scenarios, comprising 28 urban and natural scene sequences totaling 2800 pairs of images. To further evaluate the robustness and generalizability of our proposed method across different spectral bands, we additionally tested it on several publicly available visible-to-long-wave infrared (VIS–LWIR) datasets, namely DroneVehicle [52], TNO [53], M3FD [54], and FMB [55], to verify its performance under conditions free of hardware-specific characteristics.

**Mask Settings:** For quantitative evaluation on the T2SL FPA dataset, we simulated large-area blind-pixel clusters caused by random manufacturing defects in either the MW or SW band by overlaying real blind-pixel masks onto normally imaged regions. As shown in Figure 6c, the real blind-pixel mask of detector #H317 contains multiple defective pixel clusters (DPCs); we extracted five representative DPCs and depict their shapes in Figure 6d. These DPCs exhibit irregular shapes and are located at various positions across the detector array with bounding-box sizes ranging from 20×19 to 70×64 pixels. To build a simulated corrupted–reference image pair, one DPC pattern is randomly selected and overlaid onto an intact image region, while the original region serves as the ground-truth reference. To avoid artificially favoring the restoration by placing defects in homogeneous backgrounds, we follow the common practice in defect-simulation benchmarks and overlay each DPC onto image regions with noticeable local pixel variation. To enrich data diversity, each selected DPC is further augmented with a random scaling factor uniformly drawn from [0.8,2] and a random rotation by a multiple of $45^{\circ }$; the resulting mask size in the simulated dataset can therefore reach up to 140×128 pixels. Through random selection among the five DPC shapes combined with the above scaling and rotation augmentations, we generate a large and diverse set of simulated pairs. The induced defect severity, defined as the ratio of corrupted pixels to the total image area, scales with the DPC dimensions and thus spans a broad range of large-area defect conditions.
**Implementation Details:** Our proposed framework and all comparison methods were tested on a workstation equipped with an Intel Core i5-13600K (Intel Corporation, Santa Clara, CA, USA) central processing unit (CPU) running at 3.50 GHz, 32 GB RAM, and an NVIDIA GeForce RTX 2080 Ti graphics processing unit (GPU; NVIDIA Corporation, Santa Clara, CA, USA), running the Windows 11 operating system. To ensure rigorous and fair comparison, all image data underwent normalization processing. Our method employs a five-level pyramid iteration with a 7×7 patch size. The key hyperparameters are set as follows: appearance weight $\lambda_{\mathrm{app}}=0.5$, regularity weight $\lambda_{\mathrm{reg}}=10$, texture weight $\lambda_{\mathrm{tex}}=20$, proximity weight $\lambda_{\mathrm{prox}}=1$, neighborhood weight $\lambda_{\mathrm{nbr}}=5$, edge alignment scale α=1/9, and thermal preservation factor β=0.5. Hyperparameter settings are partly inherited from the corresponding prior literature [17,51] and partly determined by our own experiments: the weights of the self-proposed appearance, regularity, and texture costs ($\lambda_{\mathrm{app}}=0.5$, $\lambda_{\mathrm{reg}}=10$, $\lambda_{\mathrm{tex}}=20$) were tuned on a small held-out subset, while the proximity, neighborhood, and edge-alignment terms follow their literature defaults. Each weight balances one cost in the overall loss: $\lambda_{\mathrm{app}}$ controls pixel-level appearance fidelity, $\lambda_{\mathrm{reg}}$ regularizes the smoothness of the offset field, $\lambda_{\mathrm{tex}}$ weights the high-frequency texture guidance, and $\lambda_{\mathrm{prox}}$ and $\lambda_{\mathrm{nbr}}$ govern the spatial-proximity and neighborhood-coherence penalties. A sensitivity analysis of the three self-proposed weights and the rationale for their chosen values are provided in the Supplementary Materials (Tables S6–S12). We note that the optimal values are scene-dependent and can be re-tuned per application. We reproduced DSL and DIP following methods described in their original literature [18]. For the method by Huang et al. [17], we used official code with default parameter settings. For deep learning-based inpainting methods (PEIPNet [31] and ZITS++ [35]), we employed official codes with their default configurations. All baseline experiments performed blind pixel cluster correction under identical hardware configuration, simulated defects, and datasets.
**Evaluation Metrics:** To analyze restoration results from our proposed method and other models, following industry standards, we adopted the following metrics for a quantitative evaluation of reconstruction performance. We group them into two complementary categories. The first group assesses signal-level and structural fidelity: **PSNR** [56] measures the pixel-level mean squared error between restored and ground-truth images in decibels, and it was included as the most standard fidelity indicator; **SSIM** [56] quantifies structural similarity by comparing luminance, contrast, and structure, and it was included to capture the structural degradation that PSNR misses; **VIF** [57] evaluates the fidelity of the restored image’s visual information relative to the original, and it was included to account for human-visual-system-relevant information loss; **CC** measures the Pearson correlation between restored and ground-truth intensity distributions, and it was included to assess global radiometric consistency, which is critical for dual-band infrared applications. The second group assesses perceptual quality: **LPIPS** [58] measures the distance between restored and ground-truth images in a deep feature space learned from human judgments, and it was included to evaluate the perceptual realism that pixel-level metrics cannot capture; **FID** [59] measures the distributional distance between sets of restored and ground-truth images in Inception feature space, and it was included to evaluate whether the restored content matches the statistical distribution of real imagery.

All objective experiments were computed under identical simulated defects and datasets.

### 4.2. Ablation Studies

To thoroughly validate the effectiveness and necessity of each core component in our method, we conducted a series of ablation experiments on the SW/MW T2SL FPA dataset. This section compares five different method configurations: Full (the complete method with all guidance information), w/o Tex (removing texture guidance), w/o Edge (removing texture and edge guidance), w/o Spat (removing texture, edge, and spatial guidance), and w/o App (further removing appearance guidance). It is worth noting that since texture feature extraction and guidance depend on pre-existing edge structure generation, and edge/texture priors equally require establishment based on spatial and plane guidance, our experimental design employs a progressive removal approach to ensure the orthogonality of each guidance information.

Table 2 presents quantitative test results for different method configurations, where “↓” indicates lower is better and “↑” indicates higher is better. From the quantitative results in Table 2, it can be observed that with the progressive introduction of each guidance module, all objective evaluation metrics show a steady upward trend, fully demonstrating the effectiveness of our proposed multi-level feature-guided strategy. In the extreme case where all major guidance information is removed (w/o App), the model’s restoration capability exhibits significant degradation with the worst performance: PSNR reaches only 20.87, SSIM drops to 0.6819, VIF is merely 0.1713, and CC falls to 0.5618, while LPIPS and FID metrics reach their highest values at 0.0210 and 19.42, respectively, indicating that generated images at this stage suffer from large pixel-level errors, severe structural deficiencies, and poor perceptual quality based on deep features.

With the progressive introduction of appearance (App), spatial (Spat), and edge (Edge) guidance, metrics show stepwise improvements (e.g., PSNR steadily increases from 20.87 dB to 22.95 dB). This demonstrates that spatial layout and edge prior information can effectively constrain the image macro-structure, helping the model establish correct geometric topological relationships within missing regions. Of particular note is that when texture (Texture) guidance is finally introduced into the complete model (Full), the model performance achieves its most significant leap. Compared to w/o Tex, the complete method’s PSNR improves by over 2 dB (reaching 25.03 dB), while the SSIM increases to 0.8405, and the LPIPS decreases to 0.0108. This significant gain directly demonstrates the core and irreplaceable role of texture feature guidance in recovering high-frequency details, eliminating blur artifacts, and enhancing final visual fidelity.

**Figure 7:**
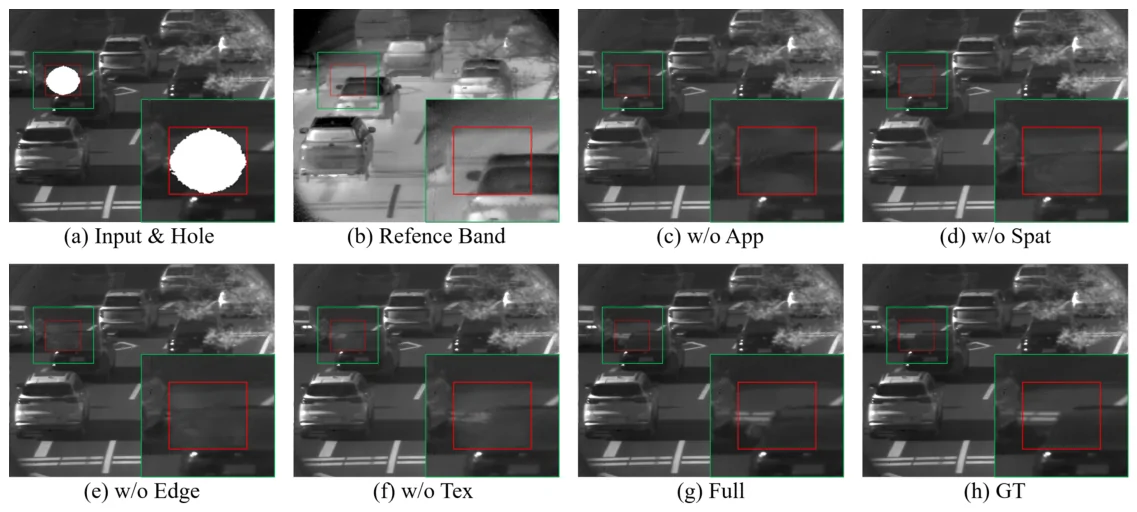
Ablation study on the “Road” sample from the SW/MW T2SL FPA dataset. (**a**) MWIR image with defective pixel clusters; (**b**) SWIR reference image; (**c**) w/o Appearance; (**d**) w/o Spatial; (**e**) w/o Edge; (**f**) w/o Texture; (**g**) Full model; (**h**) GT. Colored rectangular boxes highlight regions of interest for better visual comparison.

Figure 7 intuitively demonstrates the visual restoration effects of different configuration methods in an actual complex traffic scene, where blind pixel clusters cover partial road markings and vehicle body structures in the upper-left region. Through observing restoration effects on vehicles and road markings, quantitative metric differences among configurations are fully corroborated in visual details. In early ablation configurations (w/o App and w/o Spat), due to the lack of strong edge and texture constraints, the method struggles to infer correct semantics for missing regions. For instance, in road marking restoration, these two methods produce markings with vanished shapes and fractured edges, while vehicle structures exhibit severe blur effects failing to naturally transitions with surroundings.

With the addition of spatial and edge guidance (w/o Edge and w/o Tex), the model’s perceptual capability for image structure significantly enhances. In vehicle part restoration, the geometric contours of vehicles begin to become clear; straight edges of road markings also receive preliminary correction and connection. Finally, comparing w/o Tex and Full methods reveals that although models lacking texture guidance can recover partial structures based on structural guidance information, restored areas appear overly smooth and blurry, lacking realism. The complete Full method, under texture prior guidance, not only accurately completes the complex structural lines of vehicles and sharp edges of road signs, it also generates texture details highly consistent with real images within restored regions. Final restoration results achieve seamless visual integration with surrounding backgrounds.

### 4.3. Evaluations on Datasets

As shown in Table 3, we present quantitative comparisons between our proposed PRISM and five representative baseline methods across five datasets: SW/MW T2SL FPA, DroneVehicle [52], TNO [53], M3FD [54], and FMB [55]. These baselines include dual-band correction algorithms (DIP and DSL), state-of-the-art learning-based inpainting networks (PEIPNet and ZITS++ ), and structure-guided inpainting methods. The best and second-best results for each metric are highlighted in bold and underlined, respectively. Detailed per-dataset quantitative comparisons are provided in Tables S1–S5 of the Supplementary Materials.

**Figure 8:**
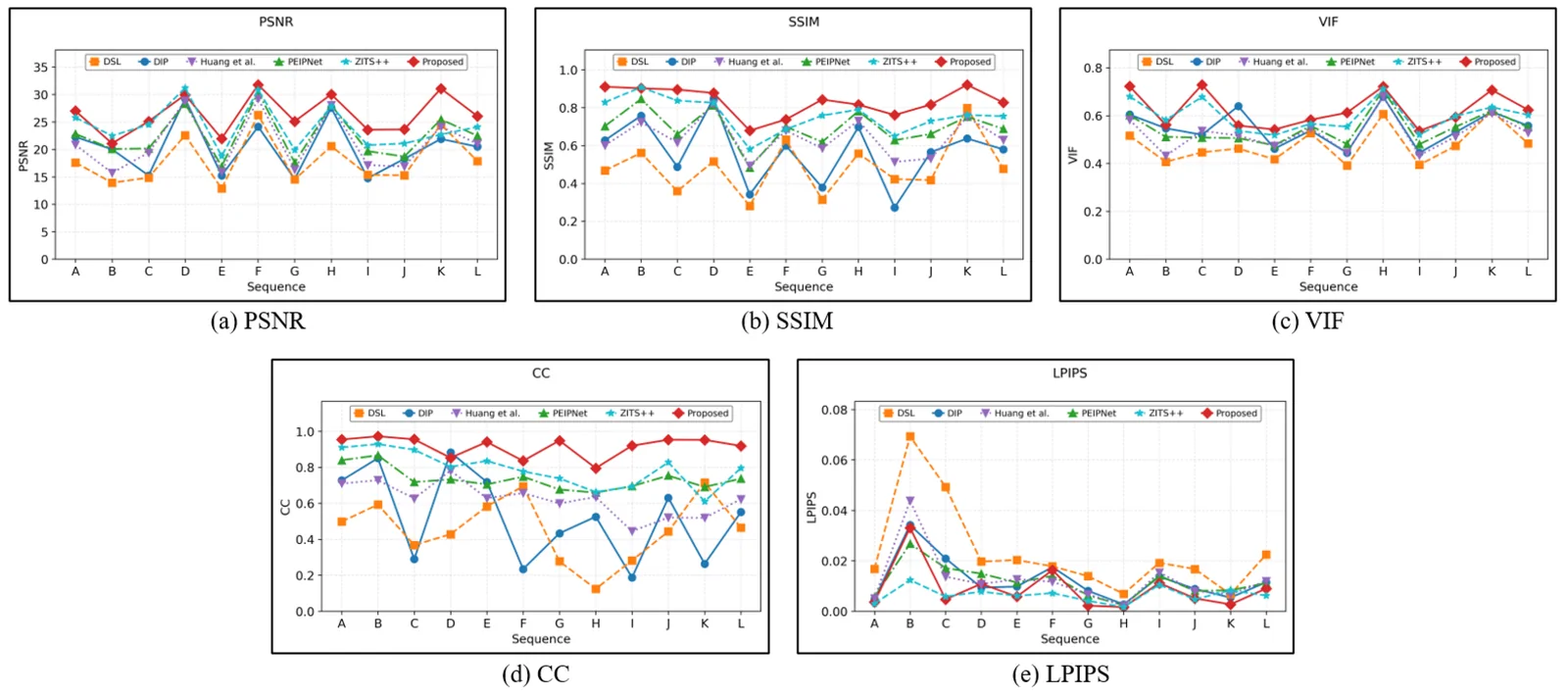
The average of the five metrics on the 12 images sequences from the all datasets. The five methods such as DSL [18], DIP [18], Huang et al. [17], PEIPNet [31] and ZITS++ [35] are used for comparison. Proposed denotes our proposed method, PRISM.

Our method achieves top performance across all fidelity metrics: PSNR (26.06 dB), SSIM (0.8266), VIF (0.6238), and CC (0.9191), while ranking second on perceptual metrics LPIPS (0.0090) and FID (8.56), which is slightly behind the results of the generative network ZITS++. Regarding the selection of comparison methods, PEIPNet and ZITS++ represent the most recent advances in deep learning-based image inpainting at the publication date of this paper, making them appropriate choices for evaluating our method against contemporary state-of-the-art approaches. Fine-grained results for different scene sequences within datasets are shown in Figure 8, where A–L correspond to 12 evaluation sequences (FID is a distribution-level metric; only the global mean is reported).

Specifically, compared to the strongest deep learning baseline ZITS++, PRISM achieves improvements of 1.96 dB, 0.0719, 0.0209, and 0.1232 in PSNR, SSIM, VIF, and CC, respectively, demonstrating superior pixel-level fidelity and structural consistency. Compared to the structure-guided restoration baseline (Huang et al. [17]), PRISM further achieves improvements of 5.02 dB in PSNR and 0.2968 in CC. For dual-band blind pixel cluster correction algorithms, compared to DIP and DSL, PRISM’s PSNR improves by 5.56 dB (20.50→26.06 dB) and 8.20 dB (17.86→26.06 dB), respectively, reducing the FID from 23.93 and 14.72 to 8.56 and the LPIPS from 0.0115 and 0.0225 to 0.0090. The above significant performance advantages are primarily attributed to the proposed Cross-band Multi-level Feature Analysis module: this module can extract local edge and texture priors cross-modally at multiple scales while accurately capturing spatial prior information with long-distance contextual features globally. It is worth noting that PRISM is slightly inferior to ZITS++ on perceptual LPIPS and FID metrics; this mainly stems from our method’s framework foundation as traditional patch-based restoration relying on the searching for and copying of local textures within images, thus lacking the inherent capabilities of generative models (such as ZITS++) in fitting large-scale real image distributions and performing global semantic inference.

**Figure 9:**
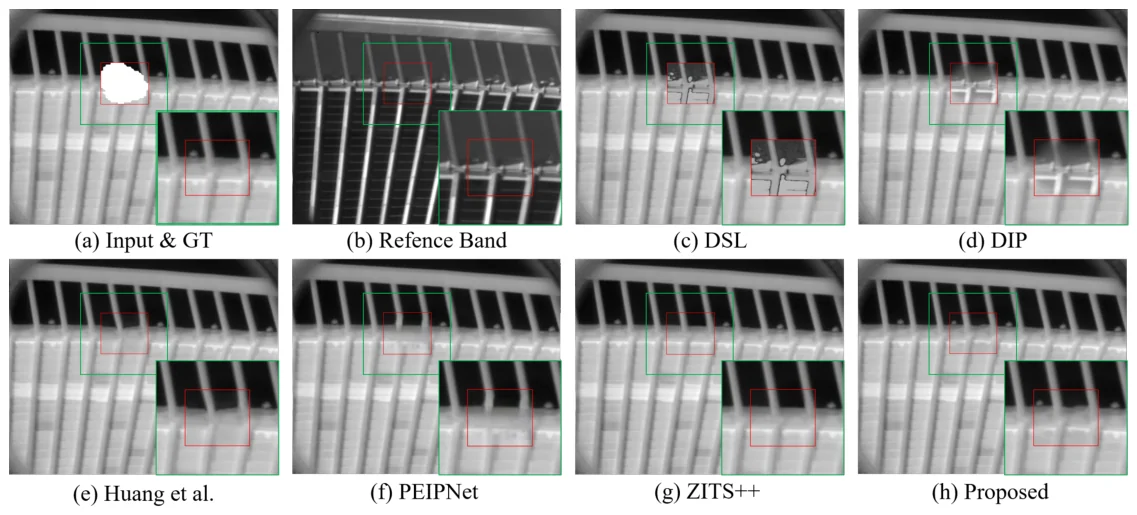
Qualitative comparison on the “Architecture” sequence from the SW/MW T2SL FPA dataset. (**a**) MWIR image with defective pixel clusters; (**b**) SWIR reference image; (**c**–**h**) inpainted results of DSL [18], DIP [18], Huang et al. [17], PEIPNet [31], ZITS++ [35], and the proposed PRISM, respectively. Colored rectangular boxes highlight regions of interest for better visual comparison. The magnified inset in (**a**) shows the ground-truth pixels corresponding to the green-box region.

Due to space limitations, we present the qualitative comparison results from four challenging scenarios selected from our datasets. Figure 9 illustrates an architectural scene with significant radiance differences and intersecting planes of steel and glass mixtures from the SW/MW T2SL FPA dataset. The primary challenge here lies in blind pixel clusters covering multiple intersecting slender lines located at intersections of two crossing named structures, requiring the algorithm to accurately reconstruct such complex repetitive textures.

**Figure 10:**
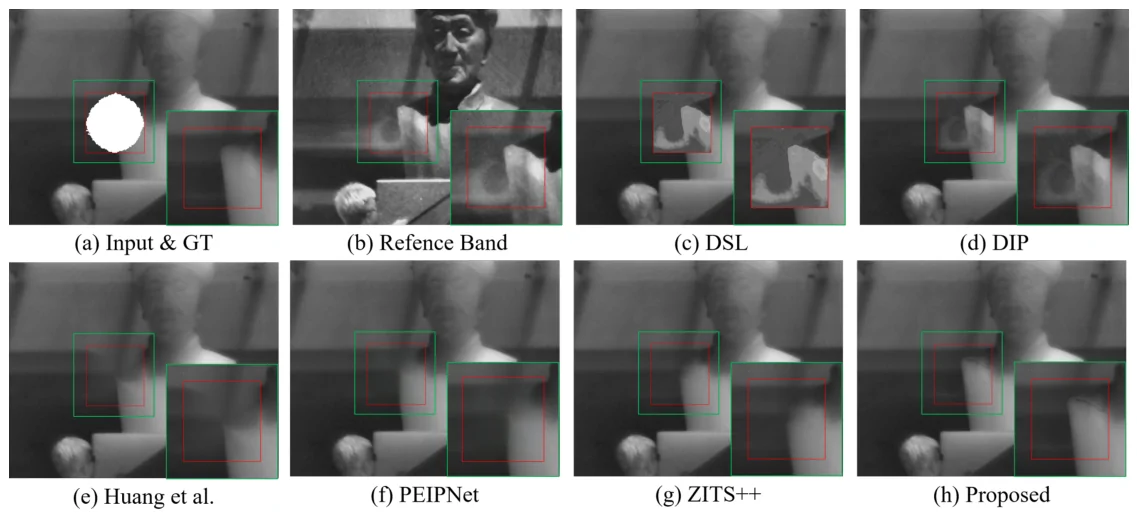
Qualitative comparison on the “Stone sculpture” from the SW/MW T2SL FPA dataset. (**a**) MWIR image with defective pixel clusters; (**b**) SWIR reference image; (**c**–**h**) inpainted results of DSL [18], DIP [18], Huang et al. [17], PEIPNet [31], ZITS++ [35], and the proposed PRISM, respectively. Colored rectangular boxes highlight regions of interest for better visual comparison. The magnified inset in (**a**) shows the ground-truth pixels corresponding to the green-box region.

Figure 10 depicts a stone sculpture scene from the SW/MW T2SL FPA dataset where reference band imaging exhibits poor quality and severe non-uniformity noise interference with blind pixel clusters located at shoulder contour positions. The challenge in this scenario lies in selectively utilizing reference band information while naturally recovering smooth surface transitions and sharp object boundaries.

**Figure 11:**
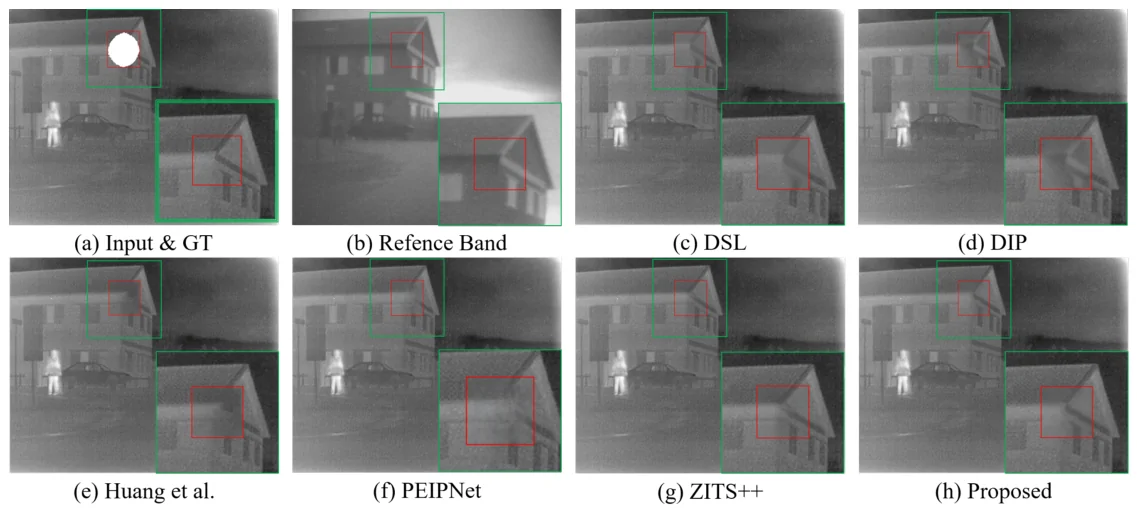
Qualitative comparison on the “Triclobs” from the TNO [53] dataset. (**a**) MWIR image with defective pixel clusters; (**b**) SWIR reference image; (**c**–**h**) inpainted results of DSL [18], DIP [18], Huang et al. [17], PEIPNet [31], ZITS++ [35], and the proposed PRISM, respectively. Colored rectangular boxes highlight regions of interest for better visual comparison. The magnified inset in (**a**) shows the ground-truth pixels corresponding to the green-box region.

Figure 11 presents an outdoor building and rural street scene from the TNO [53] dataset, presenting challenges in recovering clear building edges and geometric corners, where blind pixel clusters are located at eave junctions on house sides.

**Figure 12:**
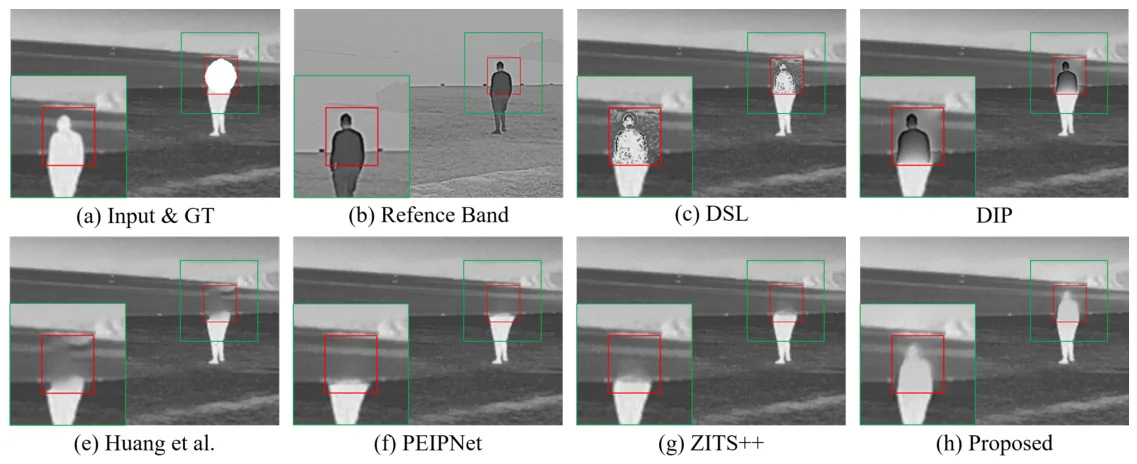
Qualitative comparison on the “Bench” from the M3FD [54] dataset. (**a**) MWIR image with defective pixel clusters; (**b**) SWIR reference image; (**c**–**h**) inpainted results of DSL [18], DIP [18], Huang et al. [17], PEIPNet [31], ZITS++ [35], and the proposed PRISM, respectively. Colored rectangular boxes highlight regions of interest for better visual comparison. The magnified inset in (**a**) shows the ground-truth pixels corresponding to the green-box region.

Figure 12 shows a pedestrian scene on an open beach from the M3FD [54] dataset, presenting challenges where large-area blind pixels completely occlude key upper body contours (head, shoulders); due to the imaging system differences between the reference and target bands, infrared contours are larger than visible light contours, making accurate human structure reconstruction highly challenging.

As specifically shown in Figure 9, when processing large-scale park art architectural structures with intersecting planes of steel and glass mixtures, maintaining temperature gradients and edge consistency is extremely challenging. When processing oblique architectural frames, PRISM employs a topology-preserving distance alignment mechanism to correct directional deviations and ensure physical authenticity in MWIR. This not only avoids the steel structure contraction caused by erroneous SWIR illumination pattern features introduced by DIP and DSL but also overcomes the structure gradient vanishing and bending problems occurring in traditional block-based restoration methods such as that used by Huang et al. while also avoiding the contextually inconsistent right-angle transformation of oblique frames produced by the deep learning method PEIPNet. The deep learning method ZITS performs excellently in handling oblique architectural frameworks but fails to recover convex structural features when processing horizontal structures; in contrast, our proposed PRISM demonstrates more balanced restoration performance.

As shown in Figure 10, when reference bands exhibit severe spectral or fixed-pattern noise (FPN) interference, both DSL and DIP algorithms show evident cross-band noise leakage with poor restoration quality. The method by Huang et al. exhibits erroneous restoration phenomena where sculpture edges blend with background edge features, failing to recover the main structure. Deep learning methods ZITS and PEIPNet produce similar restoration effects, both reasonably recovering background structures and generating relatively natural sculpture boundaries; however, due to their design inability to utilize cross-band structural information, the recovered structures deviate significantly from the original image. In contrast, the PRISM method demonstrates excellent noise robustness capable of efficiently isolating noise. This occurs because edges corresponding to reference band fixed-pattern noise cannot find corresponding anchors in the target band for migration; thus, our algorithm identifies them as reference-band-exclusive edges, and subsequent texture relabeling algorithms assign labels based on restored edges while pixel-level information from fixed-pattern noise cannot influence sampling and iterative operations. Consequently, our method accurately reconstructs portrait contour information while reasonably extending background structural frameworks.

As shown in Figure 11, when significant cross-modal radiance differences exist in rural buildings and roads, window structures restored by DSL show obvious fractures and mismatches; DIP-restored window structures exhibit significant radiance differences because these two methods establish simple mappings based on pixel relationships incapable of handling scenarios with severe cross-modal radiance differences to address modality distortion effects. Images restored by Huang et al.’s method display structural blurring and texture radiation confusion caused by the competition between eave structures on the building front and side faces. The restoration results from the deep learning method PEIPNet show obvious blur and artifacts, which also result from the error handling of structure competition phenomena. Although deep learning method ZITS++ can correctly handle eave structures, it erroneously over-extends left-side window structures, almost penetrating the entire blind pixel cluster region, causing subjective visual disharmony. Our PRISM algorithm extracts edge and texture information from reference bands while sampling from target bands during iteration; when restoring windows and eave structures, it samples image patches from nearby normal pixels with high posterior label probability, enabling the correct handling of inter-band information differences and the recovery of target band radiance intensity information without exhibiting ZITS++’s left-side window structure erroneous extension phenomenon.

As shown in Figure 12, when large-area blind pixel clusters completely occlude key upper body contours, although datasets undergo strict registration, human bodies exhibit thermal radiation and diffusion phenomena in LWIR, making LWIR contours larger than visible light contours, imposing higher requirements on restoration methods. Existing dual-band blind pixel cluster correction methods DSL and DIP show significant failure modes: DSL produces numerous noisy pixels and smooth pixels; DIP exhibits radiance mismatch, portraying originally bright human bodies as darker infrared radiation features. Images restored by Huang et al.’s method not only fail to effectively recover human body structures but also show continuous structure breakage during background restoration processes. The deep learning algorithms ZITS and PEIPNet produce similar restoration effects; although solving the continuous structure breakage problems in backgrounds that occurred in the work of Huang et al., significant coastline boundaries block the propagation of human thermal radiation features, resulting in human body structure loss. Our proposed PRISM, during blind pixel cluster reconstruction, first extracts significant structural feature information from reference bands; combined feature extraction algorithms effectively extract core pedestrian contours, successfully recovering pedestrian thermal features within blind pixel cluster regions and the natural extension of background features under conditions where other algorithms all fail, relying on cross-band adaptive information extraction and structure restoration modules.

In summary, comparison methods demonstrate common failure modes across these scenarios: traditional single-band restoration methods such as those of Huang et al. [17] struggle to effectively recover large-area missing structures and texture information, typically exhibiting boundary blurring and incomplete structure recovery problems; dual-color blind pixel cluster correction methods (DIP and DSL) cannot handle severe spectral radiance differences (such as physical differences between reflection and thermal radiation), frequently erroneously transferring modality-specific artifacts between bands (e.g., metal reflections, fixed-pattern noise), thereby causing target band thermal feature distortion; existing SOTA image inpainting methods such as ZITS++ [35] and PEIPNet [31] possess capabilities for inferring structures and deeper information from known pixels through large-scale data-driven feature learning and global semantic reasoning, but they are limited by single-band input frameworks and lack mechanisms for extracting cross-frequency physical imaging difference information, preventing effective cross-band analysis and the utilization of complementary information. Consequently, despite acceptable objective metrics, subjective results often exhibit evident structure error propagation as well as over-smoothing or hallucination artifacts. Addressing these common failure cases, the proposed PRISM algorithm demonstrates high robustness and high-fidelity restoration effectiveness. To reconstruct regular geometric lines, PRISM precisely extracts high-frequency structure priors from reference bands achieving target band geometric recovery, effectively avoiding the boundary loss and blur phenomena common in single-band methods. We also note that the deep-learning baselines’ stochastic-seed sensitivity is a limitation [60,61], whereas our training-free PRISM is not subject to such random-initialization variability.

### 4.4. Evaluations of Computational Efficiency and Resource Consumption

Table 4 compares the per-frame inference time, peak memory, and parameter count of our CPU-based PRISM against the GPU baselines PEIPNet and ZITS++. As expected, the GPU baselines are faster—0.274 s and 0.599 s versus 4.210 s on CPU for PRISM (about 15× and 7× slower)—because PRISM’s PatchMatch iterative search is compute-bound on CPU. In return, PRISM is training-free and consumes only 70.4 MB of DRAM, whereas ZITS++ and PEIPNet require 7465.8 MB and 1122.7 MB of VRAM with 111.55 M and 12.35 M parameters, respectively. Combined with its higher PSNR (26.06 dB on CPU versus 24.10 dB for GPU-accelerated ZITS++, Table 3), PRISM thus offers a favorable accuracy–efficiency trade-off for SWaP-constrained infrared platforms where GPU deployment is infeasible at the cost of a longer CPU runtime that grows super-linearly with larger defects or higher resolution.

## 5. Conclusions

By extracting and fusing edge, texture, and spatial priors, this paper proposes PRISM, which is a method for the automatic compensation of large-area blind pixel clusters in dual-band T2SL FPAs utilizing both intra-band and inter-band information. Extensive experiments conducted on our proprietary T2SL FPA dataset and public multi-spectral datasets demonstrate that PRISM significantly outperforms state-of-the-art dual-band compensation algorithms. Specifically, compared to non-deep learning methods of the same category, PRISM achieves comprehensive quantitative metric improvements; compared to the deep learning method ZITS++, although PRISM shows slightly inferior performance on perceptual LPIPS and FID metrics, it effectively eliminates the modality-specific artifacts and thermal signal distortion problems present in existing methods.

The PRISM algorithm provides a robust, training-free, and physically interpretable solution for enhancing dual-color detector device availability without risks of data-driven hallucination artifacts, though further exploration space remains. For instance, iterative patch matching and dynamic planning processes incur substantial computational overhead on standard CPUs without algorithmic optimization, typically requiring several seconds to restore one frame. However, unlike deep learning models typically requiring substantial memory bandwidth and dedicated AI accelerators at edge devices, our discrete optimization steps possess high parallelizability. In future work, we plan to optimize algorithms through exploring hardware-oriented architectures (such as FPGA parallelization) to achieve real-time processing, thereby significantly accelerating blind pixel cluster restoration processes. Furthermore, extending the spatial restoration framework to exploit temporal continuity in dual-band video sequences will be a key focus of our subsequent research.

## Figures and Tables

**Table 1:** Comparison of representative defective-pixel correction and image inpainting methods.

Method	Input Modality	Structural Guidance	Texture Guidance	Training Requirement	Cross-Band Info. Usage
DSL [18]	Dual band	No	No	No	Pixel-level
DIP [18]	Dual band	No	No	No	Pixel-level
Huang et al. [17]	Single band	Yes	No	No	No
PEIPNet [31]	Single band	No	Yes	Yes	No
ZITS++ [35]	Single band	Yes	Yes	Yes	No
PRISM (Ours)	Dual band	Yes	Yes	No	Feature-level

**Table 2:** Ablation study of different modules on SW/MW T2SL FPA dataset. In the Components columns, ✓ indicates the corresponding module is included and ✗ indicates it is removed.

Model	Components				SW/MW T2SL					
	App	Spat	Edge	Tex	PSNR↑	SSIM↑	VIF↑	CC↑	LPIPS↓	FID↓
Full	✓	✓	✓	✓	25.03	0.8405	0.3627	0.8509	0.0108	11.09
w/o Tex	✓	✓	✓	✗	22.95	0.7608	0.2550	0.6985	0.0145	14.66
w/o Edge	✓	✓	✗	✗	22.60	0.7496	0.2436	0.6741	0.0156	15.87
w/o Spat	✓	✗	✗	✗	22.33	0.7455	0.2371	0.6516	0.0159	16.56
w/o App	✗	✗	✗	✗	20.87	0.6819	0.1713	0.5618	0.0210	19.42

**Table 3:** Quantitative comparison of image inpainting performance averaged over five datasets: SW/MW T2SL FPA, DroneVehicle [52], TNO [53], M3FD [54], and FMB [55].

Metric	DSL [18]	DIP [18]	Huang [17]	PEIPNet [31]	ZITS++ [35]	Ours
PSNR↑	17.86	20.50	21.04	22.47	24.10	**26.06**
SSIM↑	0.4772	0.5796	0.6296	0.6875	0.7547	**0.8266**
VIF↑	0.4833	0.5575	0.5282	0.5513	0.6029	**0.6238**
CC↑	0.4642	0.5512	0.6223	0.7371	0.7959	**0.9191**
LPIPS↓	0.0225	0.0115	0.0119	0.0112	**0.0062**	0.0090
FID↓	14.72	23.93	12.30	13.93	**7.10**	8.56

Best results are highlighted in **bold**, and the second-best results are underlined. ↑ and ↓ indicate that higher and lower values are better, respectively.

**Table 4:** Inference efficiency comparison averaged over 50 image pairs on our datasets (image size: 640×512, defect mask: 140×128). All methods were evaluated on the same workstation. PRISM runs entirely on CPU and dynamic random-access memory (DRAM); deep-learning baselines run on GPU and video random-access memory (VRAM).

Method	Device	Runtime (s)	Peak Mem. (MB)	Params (M)
PEIPNet [31]	GPU	0.274	1122.7	12.35
ZITS++ [35]	GPU	0.599	7465.8	111.55
**PRISM (Ours)**	CPU	4.210	70.4	–

## Data Availability

The data presented in this study are available on request from the corresponding author due to restrictions related to proprietary detector systems. Partial benchmark data and code will be made publicly available upon paper acceptance.

## Supplementary Materials

The following supporting information can be downloaded at: https://www.mdpi.com/article/10.3390/s26175417/s1. Table S1: Quantitative comparison on the SW/MW T2SL FPA dataset. Table S2: Quantitative comparison on the DroneVehicle dataset. Table S3: Quantitative comparison on the TNO dataset. Table S4: Quantitative comparison on the M3FD dataset. Table S5: Quantitative comparison on the FMB dataset. Table S6: Role and selection basis of each hyperparameter. Table S7: Sensitivity analysis of $\lambda_{\mathrm{tex}}$. Table S8: Sensitivity analysis of $\lambda_{\mathrm{reg}}$. Table S9: Sensitivity analysis of $\lambda_{\mathrm{app}}$. Table S10: Per-sequence PSNR (dB)/SSIM for $\lambda_{\mathrm{tex}}\in \left\{10,20,30\right\}$. Table S11: Per-sequence PSNR (dB)/SSIM for $\lambda_{\mathrm{reg}}\in \left\{5,10,15\right\}$. Table S12: Per-sequence PSNR (dB)/SSIM for $\lambda_{\mathrm{app}}\in \left\{0.25,0.5,0.75\right\}$.
