# Supplementary material for: PRISM: Feature-Guided Hierarchical Inpainting for Dual-Band Infrared Defective Pixel Clusters
Source: Sensors (Basel). 2026 Aug 27;26(17):5417. doi: 10.3390/s26175417 (PMC13568280; doi:10.3390/s26175417)
Supplement: Supplementary file 1 [file sensors-26-05417-s001.zip › Supplementary_hyperparameters.pdf]

Hyperparameter Settings and Sensitivity Analysis

This document provides the detailed justification and sensitivity analysis for the key hyperparameters used in the proposed PRISM method, complementing the Implementation Details in the main manuscript.

1 Role of each hyperparameter

The overall loss combines several weighted costs. Each weight balances one complementary term:

Table S6: Role and selection basis of each hyperparameter.

Symbol	Value	Role in the loss / Selection basis
λ_{app}	0.5	Pixel-level appearance fidelity to the matched source patches. Determined by the sensitivity analysis in Section 2.
λ_{reg}	10	Regularizes the smoothness / coherence of the nearest-neighbor offset field. Determined by the sensitivity analysis in Section 2.
λ_{tex}	20	Weights the high-frequency texture feature guidance (recovery of fine detail). Determined by the sensitivity analysis in Section 2.
λ_{prox}	1	Spatial-proximity penalty (favors nearby valid source patches). Adopted from Huang et al. [1] with suggested default.
λ_{nbr}	5	Neighborhood-coherence penalty (robust kernel on offset differences). Adapted from Newson et al. [2], with our robust kernel; near-default strength.

2 Sensitivity analysis of the self-proposed weights

We vary each of the three self-proposed weights (λ_{app}, λ_{reg}, λ_{tex}) around its chosen value on a held-out subset of the SW/MW T2SL FPA dataset, while keeping the other weights fixed. Restoration quality is measured by average PSNR (dB) and average SSIM over the subset (higher is better). All three chosen values coincide with the observed optimum of their respective sweeps. The per-sequence PSNR and SSIM underlying these averages are tabulated in Tables 5–7 below.

Table S7: Sensitivity to λ_{tex} (other weights fixed at chosen values).

λ_{tex}	avg. PSNR (dB)	avg. SSIM
10	21.86	0.7931
20 (chosen)	**23.22**	**0.8260**
30	22.90	0.8158

3 Interpretation

For all three weights, restoration quality peaks at the chosen value and degrades smoothly on either side, indicating that the settings are near a stable optimum rather than a fragile local choice:

Table S8: Sensitivity to λ_{reg} (other weights fixed at chosen values).

λ_{reg}	avg. PSNR (dB)	avg. SSIM
5	23.08	0.8232
10 (chosen)	**23.32**	**0.8312**
15	23.05	0.8235

Table S9: Sensitivity to λ_{app} (other weights fixed at chosen values).

λ_{app}	avg. PSNR (dB)	avg. SSIM
0.25	23.1635	0.8304
0.5 (chosen)	**23.23**	**0.8327**
0.75	23.1057	0.8300

- $\lambda_{\mathbf{tex}}$ shows the strongest dependence: raising it from 10 to 20 improves both PSNR (+1.36 dB) and SSIM (+0.0329), while a further increase to 30 slightly over-emphasizes fine texture and reduces both metrics. The chosen value 20 therefore best balances detail recovery against over-texturing.
- $\lambda_{\mathbf{reg}}$ is well centered: PSNR and SSIM both peak at 10 and fall almost symmetrically at 5 and 15, confirming that the selected regularity strength keeps the offset field coherent without over-smoothing.
- $\lambda_{\mathbf{app}}$ is the least sensitive: PSNR varies within a narrow 0.13 dB band across 0.25–0.75, with the chosen value 0.5 giving the best score. This mild dependence explains why a default near 0.5 transfers robustly across scenes.

We emphasize that the optimal weights are scene-dependent: scenes with larger defect clusters or stronger radiometric gradients may favor a different balance between appearance, regularity, and thermal preservation. The values reported in the main manuscript are therefore intended as adjustable defaults; practitioners should re-tune them on a small calibration subset of their own data when deploying PRISM to a new infrared platform or defect regime.

Test results for $\lambda_{\mathbf{tex}}$

Table S10: Per-sequence PSNR (dB) / SSIM for $\lambda_{tex} \in \{10, 20, 30\}$.

Sequence	λ_{tex}	PSNR (dB)	SSIM
seq5010	10	21.4892	0.7387
seq5010	20	21.6501	0.7680
seq5010	30	21.1325	0.7464
seq5071	10	23.6788	0.8509
seq5071	20	26.4113	0.8814
seq5071	30	26.0211	0.8806
seq5079	10	20.9656	0.8613
seq5079	20	22.3122	0.8835
seq5079	30	21.9334	0.8685
seq5135	10	20.1446	0.7158
seq5135	20	21.0448	0.7541
seq5135	30	20.7951	0.7521
seq5152	10	23.0405	0.7986
seq5152	20	24.6573	0.8428
seq5152	30	24.6162	0.8314

Test results for λ_{reg}

Table S11: Per-sequence PSNR (dB) / SSIM for $\lambda_{\text{reg}} \in \{5, 10, 15\}$.

Sequence	λ_{reg}	PSNR (dB)	SSIM
seq5010	5	21.3926	0.7695
seq5010	10	21.5816	0.7768
seq5010	15	21.2134	0.7593
seq5071	5	26.2708	0.8755
seq5071	10	26.3109	0.8769
seq5071	15	25.8649	0.8759
seq5079	5	22.1473	0.8815
seq5079	10	22.2925	0.8866
seq5079	15	22.2155	0.8854
seq5135	5	21.2073	0.7574
seq5135	10	21.3621	0.7649
seq5135	15	21.0854	0.7542
seq5152	5	24.3716	0.8321
seq5152	10	25.0505	0.8506
seq5152	15	24.8947	0.8427

Test results for λ_{app}

Table S12: Per-sequence PSNR (dB) / SSIM for $\lambda_{\text{app}} \in \{0.25, 0.5, 0.75\}$.

Sequence	λ_{app}	PSNR (dB)	SSIM
seq5010	0.25	21.3519	0.7714
seq5010	0.50	21.2867	0.7712
seq5010	0.75	21.2576	0.7728
seq5071	0.25	26.7596	0.8908
seq5071	0.50	26.6998	0.8883
seq5071	0.75	26.6049	0.8859
seq5079	0.25	22.1987	0.8871
seq5079	0.50	22.2654	0.8854
seq5079	0.75	22.4544	0.9001
seq5135	0.25	20.7805	0.7518
seq5135	0.50	20.8080	0.7570
seq5135	0.75	20.8441	0.7441
seq5152	0.25	24.7269	0.8507
seq5152	0.50	25.1102	0.8615
seq5152	0.75	24.3674	0.8473

References

[1] Huang, J.-B.; Kang, S.B.; Ahuja, N.; Kopf, J. Image completion using planar structure guidance. *ACM Trans. Graph.* **2014**, *33*, 129:1–129:10.

[2] Newson, A.; Almansa, A.; Fradet, M.; Pérez, P.; Trescases, A. Video inpainting of complex scenes. *SIAM J. Imaging Sci.* **2014**, *7*, 1993–2019.
