# Supplementary material for: PRISM: Feature-Guided Hierarchical Inpainting for Dual-Band Infrared Defective Pixel Clusters
Source: Sensors (Basel). 2026 Aug 27;26(17):5417. doi: 10.3390/s26175417 (PMC13568280; doi:10.3390/s26175417)
Supplement: Supplementary file 1 [file sensors-26-05417-s001.zip › Supplementary_quantitative_results.pdf]

Per-Dataset Quantitative Comparison Results

This document provides the per-dataset quantitative results complementing Table 2 of the main manuscript, which reports the metrics averaged over the five evaluation d atasets: SW/MW T2SL FPA, DroneVehicle [5], TNO [6], M3FD [7], and FMB [8]. The same six methods as in the main manuscript are compared: DSL and DIP [1], Huang et al. [2] (StructCompletion), PEIPNet [3], ZITS++ [4], and the proposed PRISM. Best results are highlighted in **bold**, and the second-best results are underlined.

1 Per-dataset results

Table S1: Quantitative comparison on the SW/MW T2SL FPA dataset.

Metric	DSL	DIP	Huang et al.	PEIPNet	ZITS++	Ours
PSNR↑	17.5154	20.5575	20.3671	22.0825	23.9711	**25.4870**
SSIM↑	0.4930	0.6175	0.6334	0.7084	0.7803	**0.8557**
VIF↑	0.4824	0.5709	0.5242	0.5494	0.6109	**0.6284**
CC↑	0.4865	0.5879	0.6186	0.7571	0.8172	**0.9386**
LPIPS↓	0.0264	0.0125	0.0136	0.0125	**0.0070**	0.0103

Table S2: Quantitative comparison on the DroneVehicle dataset [5].

Metric	DSL	DIP	Huang et al.	PEIPNet	ZITS++	Ours
PSNR↑	12.8590	15.2484	15.9455	16.8975	18.8027	**21.9633**
SSIM↑	0.2804	0.3409	0.4936	0.4835	0.5810	**0.6785**
VIF↑	0.4172	0.4619	0.4732	0.4757	0.5180	**0.5418**
CC↑	0.5825	0.7187	0.6281	0.7049	0.8351	**0.9410**
LPIPS↓	0.0203	0.0098	0.0127	0.0114	0.0061	**0.0058**

Table S3: Quantitative comparison on the TNO dataset [6].

Metric	DSL	DIP	Huang et al.	PEIPNet	ZITS++	Ours
PSNR↑	26.2741	24.1400	29.2230	30.4507	30.8650	**31.7784**
SSIM↑	0.6305	0.5993	0.6789	0.6992	0.6850	**0.7376**
VIF↑	0.5262	0.5376	0.5466	0.5571	0.5659	**0.5826**
CC↑	0.6941	0.2343	0.6566	0.7482	0.7773	**0.8353**
LPIPS↓	0.0178	0.0175	0.0117	0.0140	**0.0072**	0.0163

In Table 5, PRISM and ZITS++ achieve the same best LPIPS value (0.0016); both are highlighted in bold.

Table S4: Quantitative comparison on the M3FD dataset [7].

Metric	DSL	DIP	Huang et al.	PEIPNet	ZITS++	Ours
PSNR↑	14.5165	14.5709	16.3280	17.5785	19.8936	**25.1089**
SSIM↑	0.3143	0.3783	0.5851	0.6187	0.7585	**0.8424**
VIF↑	0.3910	0.4449	0.4445	0.4813	0.5532	**0.6121**
CC↑	0.2773	0.4329	0.5992	0.6764	0.7382	**0.9487**
LPIPS↓	0.0139	0.0081	0.0067	0.0063	0.0041	**0.0022**

Table S5: Quantitative comparison on the FMB dataset [8].

Metric	DSL	DIP	Huang et al.	PEIPNet	ZITS++	Ours
PSNR↑	20.5820	27.5834	28.0384	28.0520	27.8248	**30.0289**
SSIM↑	0.5577	0.6974	0.7305	0.7817	0.7899	**0.8156**
VIF↑	0.6062	0.6790	0.6806	0.7063	0.7111	**0.7220**
CC↑	0.1243	0.5255	0.6346	0.6592	0.6630	**0.7951**
LPIPS↓	0.0069	0.0027	0.0022	0.0023	**0.0016**	**0.0016**

2 Note on the FID metric

The Fréchet Inception Distance (FID) [9] is a distribution-level metric: it quantifies the discrepancy between the feature distribution of a set of restored images and that of the corresponding ground-truth set, and is therefore defined over an image ensemble rather than over individual images. In our evaluation protocol, FID is computed once over the pooled evaluation set of all five datasets, since partitioning the evaluation images into individual datasets would leave too few samples per subset to yield a statistically stable distribution estimate. Consequently, no per-dataset FID value is reported in Tables 1–5; the global FID values for all six methods are given in Table 2 of the main manuscript.

References

[1] Nguyen, C.T.; Mould, N.; Regens, J.L. Dead pixel correction techniques for dual-band infrared imagery. *Infrared Phys. Technol.* **2015**, *71*, 227–235.

[2] Huang, J.-B.; Kang, S.B.; Ahuja, N.; Kopf, J. Image completion using planar structure guidance. *ACM Trans. Graph.* **2014**, *33*, 129:1–129:10.

[3] Ko, J.; Choi, W.; Lee, S. PEIPNet: Parametric Efficient Image-Inpainting Network with Depthwise and Pointwise Convolution. *Sensors* **2023**, *23*, 8313.

[4] Cao, C.; Dong, Q.; Fu, Y. Image inpainting by improving the incremental transformer on structural priors. *IEEE Trans. Pattern Anal. Mach. Intell.* **2023**, *45*, 12667–12684.

[5] Sun, Y.; Cao, B.; Zhu, P.; Hu, Q. Drone-based RGB-infrared cross-modality vehicle detection via uncertainty-aware learning. arXiv:2003.02437, 2020.

[6] Toet, A. The TNO multiband image data collection. *Data Brief* **2017**, *15*, 249–251.

[7] Liu, J.; et al. Target-aware dual adversarial learning and a multi-scenario multi-modality benchmark to fuse infrared and visible for object detection. In *Proc. IEEE/CVF Conf. Comput. Vis. Pattern Recognit. (CVPR)*; 2022; pp. 5792–5801.

[8] Liu, J.; et al. Multi-interactive feature learning and a full-time multi-modality benchmark for image fusion and segmentation. arXiv:2308.02097, 2023.

[9] Heusel, M.; Ramsauer, H.; Unterthiner, T.; Nessler, B.; Hochreiter, S. GANs trained by a two time-scale update rule converge to a local Nash equilibrium. In *Advances in Neural Information Processing Systems*; 2017; Vol. 30, pp. 6626–6637.
